# PGxDB: an interactive web-platform for pharmacogenomics research

**DOI:** 10.1093/nar/gkae1127

**Published:** 2024-11-20

**Authors:** Trinh Trung Duong Nguyen, Ziaurrehman Tanoli, Saad Hassan, Umut Onur Özcan, Jimmy Caroli, Albert J Kooistra, David E Gloriam, Alexander S Hauser

**Affiliations:** Department of Drug Design and Pharmacology, Faculty of Health and Medical Sciences, University of Copenhagen, 2100 Copenhagen, Denmark; Institute for Molecular Medicine Finland (FIMM), HiLIFE, University of Helsinki, Finland; BioICAWtech, Helsinki, Finland; BioICAWtech, Helsinki, Finland; Institute for Molecular Medicine Finland (FIMM), HiLIFE, University of Helsinki, Finland; Department of Drug Design and Pharmacology, Faculty of Health and Medical Sciences, University of Copenhagen, 2100 Copenhagen, Denmark; Department of Drug Design and Pharmacology, Faculty of Health and Medical Sciences, University of Copenhagen, 2100 Copenhagen, Denmark; Department of Drug Design and Pharmacology, Faculty of Health and Medical Sciences, University of Copenhagen, 2100 Copenhagen, Denmark; Department of Drug Design and Pharmacology, Faculty of Health and Medical Sciences, University of Copenhagen, 2100 Copenhagen, Denmark

## Abstract

Pharmacogenomics, the study of how an individual's genetic makeup influences their response to medications, is a rapidly evolving field with significant implications for personalized medicine. As researchers and healthcare professionals face challenges in exploring the intricate relationships between genetic profiles and therapeutic outcomes, the demand for effective and user-friendly tools to access and analyze genetic data related to drug responses continues to grow. To address these challenges, we have developed PGxDB, an interactive, web-based platform specifically designed for comprehensive pharmacogenomics research. PGxDB enables the analysis across a wide range of genetic and drug response data types - informing cell-based validations and translational treatment strategies. We developed a pipeline that uniquely combines the relationship between medications indexed with Anatomical Therapeutic Chemical (ATC) codes with molecular target profiles with their genetic variability and predicted variant effects. This enables scientists from diverse backgrounds - including molecular scientists and clinicians - to link genetic variability to curated drug response variability and investigate indication or treatment associations in a single resource. With PGxDB, we aim to catalyze innovations in pharmacogenomics research, empower drug discovery, support clinical decision-making, and pave the way for more effective treatment regimens. PGxDB is a freely accessible database available at https://pgx-db.org/

## Graphical Abstract

**Graphical Abstract:**
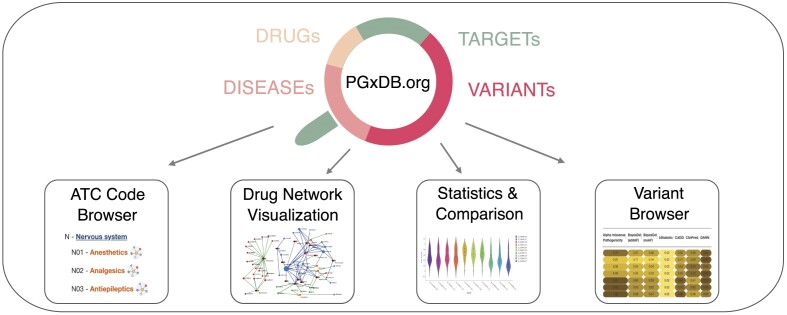

## Introduction

Pharmacogenomics (PGx), the study of the interplay between an individual's genetic profile and their response to pharmaceutical agents, represents an emerging and rapidly advancing field within personalized medicine. By elucidating the genetic factors that influence drug metabolism, efficacy, and toxicity, pharmacogenomics enables the tailoring of drug therapies to achieve optimized therapeutic outcomes, for a more precise, effective and safe drug therapy (1). This approach contrasts with the traditional ‘one-size-fits-all’ approach of prescribing drugs based on population averages independent of individual genetic markers. The integration of pharmacogenomics in clinical practice can lead to a significant reduction in adverse drug reactions, which are a leading cause of hospitalizations and deaths (2,3). Prospective genotype-guided treatment selection has been shown to reduce incidents of clinical adverse drug reactions (ADRs) with feasible implementation across diverse healthcare systems (4). This has been particularly impactful in areas such as oncology, where treatments can be customized based on the somatic genetic profile (5). Moreover, pharmacogenomics facilitates drug discovery by uncovering novel therapeutic targets and elucidating cellular pathways, while also enabling the prediction of drug efficacy and safety in different populations (6–8). By stratifying patients or subjects into groups based on their genetic profiles, pharmacogenomics can significantly reduce the rate of failures in clinical trials, leading to more efficient and targeted therapeutic development (9).

Pharmacogenomics databases and resources, like PharmGKB (10), have been instrumental in cataloging the available literature by providing comprehensive information about clinical guidelines and drug label annotations. Other notable resources in this field include the Human Cytochrome P450 (CYP) Allele Nomenclature Database (11), which focuses on variations in drug-metabolizing enzymes, and the Clinical Pharmacogenetics Implementation Consortium (CPIC) (12), offering guidelines for detailed gene/drug clinical practice guidelines. Additionally, the Pharmacogenomics Knowledge Translation (PharmCAT) interprets pharmacogenomic genotype data (13), and the NIH’s Genetic Testing Registry (GTR) provides detailed information about genetic tests (14). The ClinVar database annotates reports about human variation, interpretations of the relationship of that variation to human health, and the evidence supporting each interpretation (15). PreMedKB is a precision medicine knowledge base for interpreting relationships between indications, genes, variants and drugs (16). Finally, DAN (Drug Association Networks*)* provides a systems pharmacogenomic landscape of drug similarities based on cellular gene expression signatures (17).

PGxDB introduces a novel perspective to this array of resources by offering a highly interactive and user-friendly platform that integrates diverse data types, including molecular target profiles, adverse reactions, and indications associated with both approved drugs and investigational compounds (see Supplementary Table S1 and the case example in Supplementary Data for a comparison with other similar resources). By combining drug profiles indexed at each ATC level with detailed genetic data, health associations, and statistical associations, PGxDB delivers a more comprehensive analysis platform on the pharmacogenomics landscape. The network analysis and visualization modules further enhance the user experience, enabling an intuitive exploration of complex genetic and pharmacological interactions. This new resource complements existing databases by providing a dynamic environment for comprehensive research and hypothesis testing, thereby enriching the pharmacogenomics toolkit and facilitating association discovery and clinical applications in personalized medicine.

## Methods

### Workflow overview

**Figure 1.**
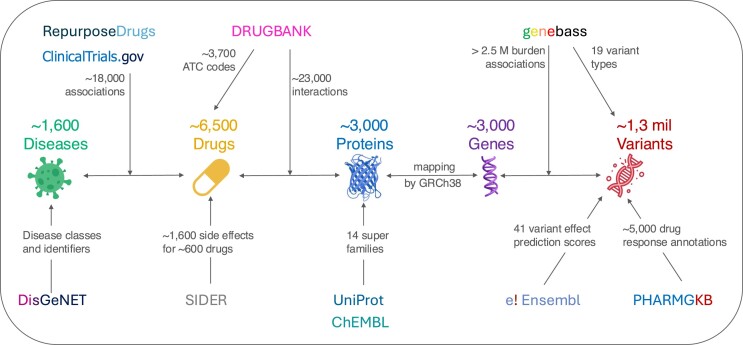
Data collection and integration workflow. The data collection and integration workflow combine diverse data types, including drugs, indications, adverse drug reactions, drug targets, genes, genetic variants and phenotype associations obtained from high-quality curated databases. It encompasses variant effect prediction scores, gene-based association summary statistics, and pharmacogenomics data. Relationships among these entities are organized using the Anatomical Therapeutic Chemical (ATC) classification system and drug-interaction data from Drugbank. The workflow highlights the source databases and includes integrated data statistics to provide a comprehensive view of the data and their integrations.

PGxDB provides a comprehensive overview of diverse data types, serving as a centralized hub for pharmacogenomics integrative analysis. The included data encompasses approved drugs and investigational compounds associated with indications target profiles, and adverse reactions. In addition, we integrated normalized variant effect prediction (VEP) scores across 41 resources, target-based drug association summary statistics, clinical guideline and drug label annotations. The menu system categorizes information by drug, indication, target, and variant search functions, enabling retrieval of relationships such as drug-target interactions, drug indication, adverse drug reactions, and variants affecting drug responses. Additionally, drugs are assigned an Anatomical Therapeutic Chemical (ATC) classification code where possible, a system maintained by the World Health Organization (WHO) (https://atcddd.fhi.no/atc_ddd_index). This classification system standardizes the categorization of medications based on their therapeutic use, pharmacological properties and anatomical site of action. The ATC system is a hierarchical classification system, structured into five levels, which serves as a browsing tool to explore drugs and investigational compounds. Figure 1 shows the overall data collection workflow, with more details provided in the section: ‘*Data collection and curation*’.

### Data collection and curation

#### Drug data

Using DrugBank database (version 5.1.12 released on 2024-03-14 for academic users) (18), we collected 6504 drug-like molecules (small molecules and biologics) interacting with one or more target proteins. Additionally, we integrated physiochemical and toxicity information for these drug-like molecules using a custom python script. Among all drug molecules, 483 are biologics (including antibodies, vaccines, recombinant therapeutic proteins or other biological molecules), and 6021 are small molecules. We further classified all drug molecules into six categories based on their clinical development and approval status (approved, vet-approved, nutraceutical, experimental, investigational, and illicit). Approved drugs are formally authorized by regulatory agencies, such as the U.S. Food and Drug Administration (FDA), the European Medicines Agency (EMA), or other national health authorities for treating human patients. Vet-approved drugs are for animal treatments whereas nutraceuticals have demonstrable nutritional effects. Investigational compounds have not been approved but are currently under investigation in clinical trials (phase I, II or III). Experimental drugs are compounds that have not yet been investigated or approved in any clinical trials but have preclinically shown to bind specific target proteins. Finally, illicit compounds are banned substances in most developed nations (such as cocaine and heroin). There are 2377 approved and 36 vet-approved drugs, 30 nutraceuticals, 1051 investigational, 2975 experimental compounds (preclinical) and 35 illicit compounds.

#### Target protein and gene annotation data

We retrieved 2969 proteins for the integrated drugs and compounds in PGxDB. We classified the protein targets into 14 superfamilies, named: ion channel, kinase, enzyme (other than kinase), transporter, GPCR, adhesion-GPCR, membrane receptor (other than GPCRs), secreted protein, structural protein, epigenetic regulator, nuclear receptor, transcription factor, surface antigen and unclassified (for proteins for which we could not find any superfamily designation). These superfamilies were originally derived from ChEMBL (version 33) (19) and were further fine-tuned manually. For instance, ‘tyrosine kinase’ was mapped to kinase and ‘7tm receptor’ was mapped to GPCR. Some of the bigger families were subdivided into smaller families, for instance, adhesion is a sub-family of GPCRs. We also integrated additional information such as the primary sequences from UniProt (20), and predicted 3D structure from the AlphaFold protein structure database (AFDB) (21). We opted to use protein structure data from AFDB over the curated structures from Protein Data Bank for better mapping due to its consistency and more comprehensive coverage in regions where experimental methods like X-ray crystallography or cryo-EM have limitations such as in flexible loops, disordered regions, or mutated residues. Finally, for each target protein, gene annotations were retrieved from the GRCh38 genome assembly (release 112, on 2024-02-20).

#### Drug-indication data

Approved and investigational drug-indication pairs are sourced from RepurposeDrugs (22), which has its own semi-automated pipeline to extract approved drug-indications via the ClinicalTrial's API (https://clinicaltrials.gov/data-api/api). Investigational indications (phase I–III) both for approved drugs and investigational compounds are extracted from the ChEMBL database (version 33) (19). Collectively, we obtained ∼1 600 unique drug-indications. We then mapped these indications to Unified Medical Language-Concept Unique Identifiers (UML-CUIs) available at (23). Next, we aggregated indications into 25 classes based on DisGeNET’s manually curated indication ontology (23). To integrate investigational drug-indications from ChEMBL, we used UniChem's API (24) to first map DrugBank IDs into standard InChiKeys and then standard InChiKeys to ChEMBL IDs. Investigational indications originating from ChEMBL are represented using Experimental Factor Ontology (EFO) (25,26] and Medical Subject Headings (Mesh) IDs (26). Consequently, we mapped all EFO and MESH IDs to UML-CUIs so that both approved and investigational indications are linked with UML-CUIs and assigned indication classes. We employed distinct color-codings for each indication class and integrated filtering options for end-users to customize the network visualization. Users can then export the visualization into a high-definition figure, which is licensed under a Creative Commons Attribution (CC BY) License, allowing to freely use, distribute, and build upon the material for scientific research and publications, provided that appropriate credit is given to the original authors.

#### Variant data

We began by obtaining single-variant and gene-based association summary statistics from Genebass (27), a repository of association statistics, to identify variants in our collected 2969 genes. Genebass provides aggregated exome-based association analyses on a wide-range of phenotypes across nearly 400 000 individuals from the UK Biobank. We selected health-related outcomes across 17 categories (Supplementary Table S2). These categories fall under the subcategory ‘First occurrences’ within ‘Health-related outcomes’ and ‘Medication’ under ‘Verbal interview,’ which are part of the ‘UK Biobank Assessment Center’ in the UK Biobank dataset (https://biobank.ndph.ox.ac.uk/showcase/). They were assigned 681 unique phenotype codes spanning a range of health outcomes from perinatal and congenital conditions to mental and behavioral disorders, infectious indications, and organ system-specific disorders. We retrieved burden association statistics including *P*-values and beta effect sizes (strength of association (and direction as standard deviation difference of the phenotype) for each gene-based and single-variant linked to these phenotypes.

With the variant markers sourced from Genebass for our collected genes and all corresponding ∼1 300 000 unique variants, we acquired corresponding variant effect prediction (VEP) scores from over 41 different algorithms including SIFT, Polyphen, PrimateAI and AlphaMissense, among others. To obtain those, we utilized two notable plugins from the Ensembl Variant Effect Predictor (28), dbNSFP and AlphaMissense (29,30). Raw prediction scores have been min-max normalized to the range [0,1] to facilitate comparison across VEPs (Supplementary Table S3 shows a list of all included algorithms).

Finally, we obtained ∼5200 clinical annotations of variant-drug interactions involving ∼420 drugs and ∼500 protein targets from PharmGKB (10). This allows users to directly compare molecular and clinical information on a by-variant basis on their drug-interacting gene of interest.

#### Adverse drug reactions data

We collected drug adverse reactions and their frequencies from SIDER (31) (http://sideeffects.embl.de/). The frequency refers to the number of patients who experienced a specific adverse reactions while taking a particular medication. This information is extracted from drug labels and is based on data available in the MedDRA (Medical Dictionary for Regulatory Activities) dictionary (32). If the percentage value for a given id had been given as an interval (e.g. 8–10), we selected the upper bound (10, in this case). Of note, the frequency is not necessarily related to the number of studies that reported the adverse drug reaction. Instead, it represents the proportion of patients in a study who experienced adverse reactions. This information is useful for understanding the prevalence of adverse reactions associated with a particular medication and can help inform clinical decisions about its use.

### Technical implementation

A number of frameworks and tools were utilized to build PGxDB. Specifically, we used Python-based packages (Pandas (33), Numpy (34), Hail (https://github.com/hail-is/hail/releases/tag/0.2.13), pyspark (https://spark.apache.org/docs/latest/api/python/), psutil (https://github.com/giampaolo/psutil) for data collection and processing. For the backend, we adopted the Django framework and for the frontend a combination of HTML, CSS and JavaScript is used. PostgreSQL database management system (DBMS) is used for storing and managing the data. It is worth noting that when construcing the relational database, we tried to keep unique identifiers of data points. This ensures that one can use the international unique identifiers of these objects—e.g. DrugBank IDs for drugs or UniProt accession numbers for proteins. For data points with more than one international identifiers, we believe that it is not optimal to attempt to convert variant identifiers across different genome annotation databases, such as Ensembl, UCSC, Gencode, RefSeq, and NCBI. We then used the Ensembl annotation method to denote variant identifiers, as it is straightforward: Chromosome_Coordinate_Reference/Alternative allele, e.g. 20_50 581 449_C/G. This annotation method is also used by Genebass. For variants with different annotation methods, such as the RefSeq variant annotation used by PharmGKB, we kept them in their original form. For the data browsers, which include the variant effect prediction scores, gene association-based summary statistics, we implemented the DataTables.js module (https://datatables.net) along with yadcf.js (https://yadcf-showcase.appspot.com). These modules facilitate sorting and filtering functionalities. For the network comparison and analysis, we used Networkx (https://networkx.org/), a Python library. The network visualizations and plots were crafted in JavaScript, with a primary emphasis on the D3.js framework (https://d3js.org) to create SVG figures and animations. We also employed 3Dmol (https://3dmol.csb.pitt.edu/), another Javascript library to build the 3D protein structure viewer. PGxDB adheres to the FAIR principles (Findable, Accessible, Interoperable, and Reusable), ensuring the data is available to a wide range of users, regardless of their technical expertise.

### Comprehensive data access and navigation tools

PGxDB provides an integrated suite of features designed to enhance data retrieval and navigation for users. Key functionalities include:

*Dedicated search pages*: Each main data type—drugs, indications, target proteins and variants—has a dedicated search page. Users can search for specific entry by entering their names or identifiers, with auto-complete suggestions to facilitate quick access. The search results provide detailed information and include hyperlinks to both internal pages and external resources such as DrugBank, UniProt, and Clinicaltrials.gov.

*Interlinked data of different types*: Drugs, proteins, genes, variant, etc., are interconnected across the website, allowing users to seamlessly navigate between related data points (detailed in Supplementary Table S4).

*Chromosome/contig name mapping tool*: To address inconsistencies in chromosome/contig naming conventions across different resources, we provide a ID-mapping tool. This tool assists users in mapping ids and names across UCSC, Ensembl, Gencode, RefSeq, and NCBI for human genome assemblies GRCh37 and GRCh38, streamlining the comparison process.

*User support and documentation*: A comprehensive tutorial page guides users on how to use specific menus, sub-menus, and functions, ensuring efficient data retrieval. Additionally, a detailed documentation page explains the website's structure and data content, aiding users in understanding and navigating the platform effectively.

*Application Programming Interface (API)*: Using the Django REST framework, our APIs allow retrieval of all underlying data using simple Python code snippets. This enables users to integrate the data into their applications and workflows efficiently or to continue local analysis.

## Results

### Protein variant lookup and interpretation

**Figure 2.**
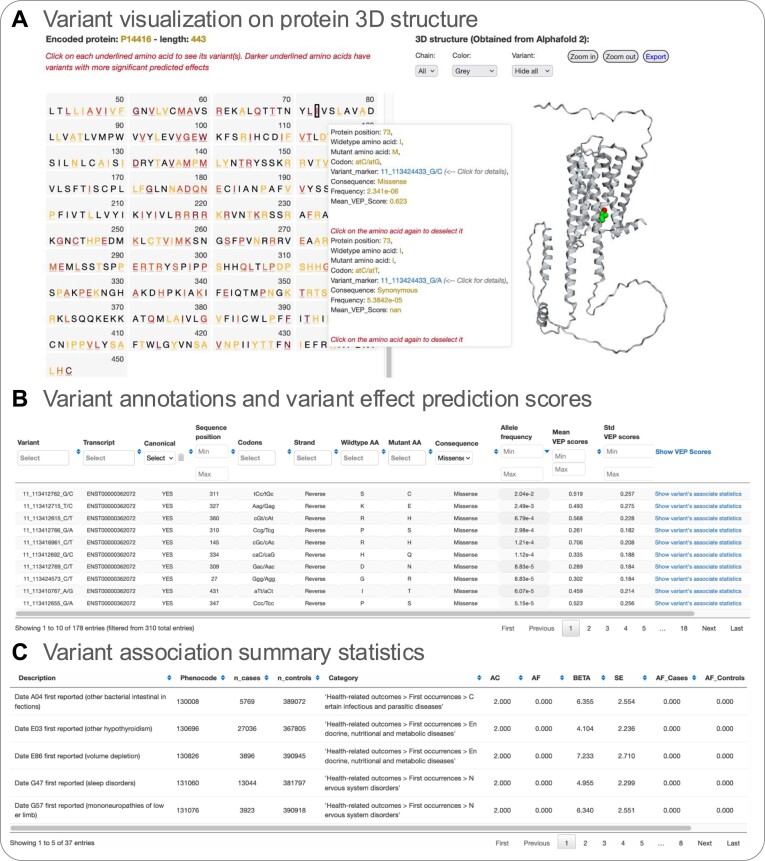
Gene-specific variant browsing tool. **(A)** Protein amino acid sequence and variant visualization on a predicted 3D protein structure **(B)** Variant annotations and linked violin plots summarizing variant effect prediction scores from 41 different algorithms (see Supplementary Table S3 for details). **(C)** Variant association summary statistics on health and medication outcome phenotypes.

This gene-specific browser offers a comprehensive tool for exploring genetic variants and their potential impacts on drug-interacting proteins. By mapping each variant to its respective protein residue, it allows users to visually interpret variant effects through interactive, color-coded displays, dynamic tables, and detailed annotations, supporting deeper insights into the functional implications of genetic variability. Each target lookup (https://pgx-db.org/target_lookup/) presents comprehensive variant information for each drug-interacting protein. The canonical protein sequence of the selected gene is shown, where each coding gene variant is mapped to the corresponding protein residue. Selecting a specific amino acid will display all variants that occur at the given position. Amino acids are color-coded to reflect the predicted effect of occurring genetic variants. Variants can be highlighted, which are then displayed in the 3D structure predicted by AlphaFold2 on the right. The structural representation can be rotated, zoomed in/ out, and exported as an image (Figure 2A). Basic variant annotations such as gene location, primary transcript, sequence position, strand location, wild type and mutant amino acids, minor allele frequency, and consequence annotations are provided in a dynamic table below (Figure 2B).

**Figure 3.**
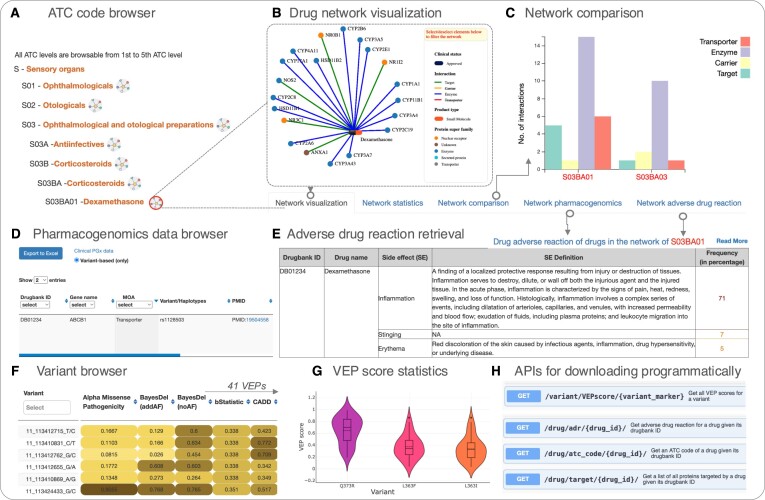
PGxDB snapshot. **(A)** Tree-like browser that allows for selection of ATC codes at all levels, from Anatomical group (level 1; e.g. B—blood and blood-forming organs) to Chemical substance group (level 5, e.g. S03BA01). Clicking on the network button next to each ATC code description opens a network detail in the right panel. **(B)** Network visualization of the drug-target interactions and drug-indication associations for the drug Dexamethasone (ATC: S03BA01) and additional tabs that provide tools and data underlying the network. Within each tab, a link to the documentation page is provided presenting more detailed explanations of features and functions. **(C)** Network feature comparison for two different ATC codes (e.g. distributions of drug-protein interaction modes in the networks of S03BA01 and S03BA03). **(D)** Pharmacogenomics data browser on clinical drug label annotations related to all drug-target interactions within the network and **(E)** Table displaying adverse drug reactions of drugs within the network (both for Dexamethasone). **(F)** Variant browsing tool for a selected gene with variant effect prediction scores from 41 distinct algorithms in color gradient ranging from 0 to 1 alongside **(G)** representations of the distributed scores of all effect prediction scores for user-selected variants **(H)** Selected examples among 15 API endpoints allowing for automated data access and testing.

The table is further extended with commonly used and readably available variant effect prediction scores (for now 41 distinct algorithms) designed to assess the potential impact of genetic variants (see Supplementary Table S3). The displayed normalized rank scores between 0 and 1 represent the ratio of the rank of the score over the total number of scores given by that algorithm. This facilitates direct comparison between different algorithms and relative interpretation. A rank score close to 1 indicates a predicted mutation to be highly impactful, regardless of the original scoring scale (Figure 3F). We also provide the mean and standard deviation across all VEP scores for quick sorting and overview (Figure 3). When choosing ‘Hide VEP scores" (top right), a link to single-variant association statistics will replace the VEP scores. A new window will display significant associations (*P*-values ≤ 0.05) of this variant when available (described in the next section). The table browser facilitates easy navigation, filtering, and sorting of variant information, with options to download the underlying data. Additionally, the accompanying chart displays interactive violin plots of effect prediction scores for all selected variants to allow easy comparison, which users can download for further reference (Figure 3G).

### Variant-based association summary statistics tool

VEP tools, which are aimed at the prediction of the pathogenicity of genetic mutations given underlying training on disease data and evolutionary conservation, are not specifically designed for the prediction of drug effects (35). Therefore, we employed additional data from large-scale population cohorts and associations to medication use and other health outcomes. This may further aid the interpretation of predicted variant impacts through their relevance to human indications and traits. We are providing summary association statistics sourced from Genebass (27) underlying the UK Biobank cohort for each coding variant for a selected protein and provide information on the population frequencies as well as the number of cases and controls for the specific variant-phenotype combination. This is provided in a pop-up browser further allowing filtering, sorting and downloading of variant-based association data (Figure 2C).

Collectively, the integration of variant effect prediction scores from multiple algorithms, along with population-based association data, allows researchers to assess variant pathogenicity within a clinical context, helping to identify genetic markers that may correlate with patient-specific drug responses.

### Heterogeneous drug networks, grouped by therapeutic, pharmacological and chemical properties

The platform offers a unique approach to combining the hierarchical ATC classification with network-based drug interaction data in a comprehensive manner. The ‘*ATC classification hierarchy browser*’ page at https://pgx-db.org/atc_lookup simplifies the search process for ATC codes. It follows a 5-tier structure, starting from the anatomical group and progressively narrowing down through therapeutic, pharmacological, and chemical levels to the specific 7-character chemical substance codes (Figure 3A). The number of unique ATC codes at each level is 14, 93, 263, 884 and 5 368, respectively. By clicking any ATC code, users can explore a network that visualizes drug-protein interactions and/or drug-indication associations.

It is additionally essential to describe the network of ATC code X, focusing on the drugs it contains. This network includes all drugs associated with the chemical substance codes at the most specific level within X’s hierarchy. For example, the ATC code ‘A07A’ represents a pharmacological group that consists of two chemical subgroups: A07AA and A07AB. A07AA contains 15 substance codes, while A07AB contains 3 substance codes. Therefore, the network of A07A includes drugs linked to all 18 of these specific substance codes. In addition to these drugs, the network also comprises the proteins that interact with them and the diseases for which drug-disease association studies exist. The interactive features of these networks are further described in the following sections.

## Analysis tools based on heterogeneous drug networks

### Network statistics

The first element users will notice is an overview table that lists drugs linked to the selected ATC code and its related subcodes. This table highlights interacting protein targets color-coded by their type of interaction (target, transporter, carrier and enzyme), as well as associated indications for each drug. Directly beneath the overview table is a panel with five tabs. The first tab, ‘Network visualization’, provides a tripartite network visualization with drugs, targets, and indication nodes (Figure 3B; described in more detail in ‘*Interactive indication-drug-protein interaction network*’ below). In the second tab, ‘Network Statistics’, overall network statistics of the selected network comprising the drugs, protein targets, and indications are presented. It also includes information on molecule types, the included agents’ clinical developmental statuses, the type of drug-protein interaction, phases of clinical trials for drug-indication associations and indication classes within the network.

### Network analysis and comparison tool

The ‘Network analysis and comparison’ tool in the third tab allows users to either analyze the network topology of the currently selected ATC code (without requiring an additional ATC code) or perform a side-by-side comparative analysis by entering another ATC code. Previous studies have shown that representing this data in a graph (network) topology can provide valuable biological insights. For example, it has been shown that targets of approved drugs tend to demonstrate higher protein-protein interaction network centrality than targets of drugs at earlier approval stages, suggesting that network centrality may be associated with therapeutic targetability (36). Moreover, it has been stated that the degree (number of connections of a node in a network), and betweenness centrality (the fraction of shortest paths through a given node in a network) are among important measurements of biological networks (37). We therefore provide a variety of these and additional network statistics to facilitate analysis and comparison options (see Figure 3C). Supplementary Table S5 provides detailed description about these comparison options.

### Pharmacogenomic insights

The next tab, ‘Network Pharmacogenomics’, presents both ‘burden data’ on gene-based and variant-specific association statistics and ‘Clinical PGx Data’ from annotated drug label information on all the drug-protein interactions within the network. When available, the ‘Clinical PGx Data’ (Figure 3D) subsection provides detailed variant annotations, including variant identification, drug mode of action, phenotype categories (e.g. efficacy or safety), clinical significance, direction of effect, associated p-values as provided in the primary studies, and included ancestries as ‘biogeographical groups’ from the discovery cohort. The ‘Burden data’ subsection displays results from burden tests, which assess the aggregate impact of genetic variants on genes (proteins) in relation to the phenotypes (drugs) within the network. This data is further categorized into gene-based and variant-based statistics, for which both statistical significance (p-value) and strength of association (BETA effect size) are presented derived from burden tests. For each association pair, up to four functional annotation categories can be selected: predicted Loss-of-Function (pLoF), missense|LC (including low-confidence pLoF variants and in-frame insertions or deletions), synonymous, and the combination pLoF and missense|LC group. Additionally, the underlying number of cohort cases and controls are presented.

This allows researchers to investigate whether specific genetic variants and/or gene-based associations for specific protein groups contribute to the likelihood of experiencing certain ADRs and drug associations, directly linking genetic burden testing results to real-world adverse effects. This allows for a more comprehensive analysis of how drug–protein interactions within the network are influenced by genetic variation guiding more precise drug discovery and treatment strategies.

### Adverse drug reaction overview

The last tab, ‘Network adverse drug reaction’, shows adverse reactions, if available, for drugs within the network (Figure 3E). For each adverse drug reaction (or side effect), a short definition and the frequency found in the surveyed population are presented. A gradient color coding is applied to quickly provide an overview of more frequent (*red*) and less frequent (*orange*) adverse effects.

This feature highlights drugs with frequent adverse effects, which can help researchers prioritize future pharmacogenomic studies. By identifying drugs with high variability in ADRs, researchers can focus on those medications and their targets for investigation of genetic markers via the ‘target’ and ‘variant’ tabs aimed at uncovering possible genetic predispositions.

### Interactive indication–drug–target interaction network

**Figure 4.**
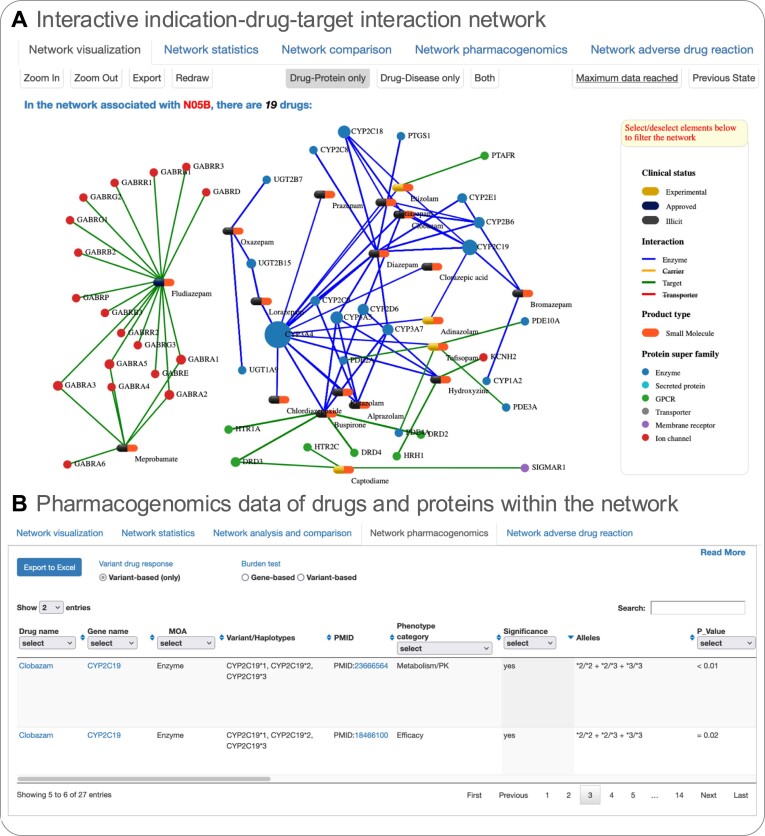
Network snapshot. **(A)** Interactive indication-drug-protein interaction network of N05B (Anxiolytics) showing the relationships of the three nodes: drugs (pill-shaped), targets (round), and indications (triangular). In this case, the ‘Drug-Protein only’ network is selected. The interactive legend panel allows node filtering and manipulation (e.g. changing node and edge colors). The button set allows zooming, redrawing, and exporting the visualization in different formats. Of note, larger networks require data loading in chunks to avoid overpopulation of the network. This can be retrieved via the ‘Get more data’ button in the top right and switches to ‘Maximum data reached’ if the full network is visible. **(B)** Pharmacogenomics profile underlying variant annotations reported as an association between a variant (e.g. SNP, indel, repeat, haplotype) and a phenotype category (e.g. metabolism/pk or toxicity) from a single publication of drugs and proteins within the N05B network.

We developed an interactive indication–drug–target interaction network that allows researchers to visually explore and analyze complex relationships between indications, drugs and their molecular targets facilitating the identification and rationalization of on- and off-target-based drug response variabilities. The more than 6000 interactive tripartite networks offer a dynamic view and contrastive comparison across any ATC-level indication-drug-target interactions (Figure 4A).

Each node in the network represents either a drug (pill-shaped, distinguished by molecule type and clinical status), a protein (round, color-coded by superfamily), or indication (triangular, color-coded by indication class). Expanding a node reveals additional details such as protein properties, drug molecule structure, aliases, drug indication and development stage.

Links represent drug-protein interactions and/or drug-indication associations. Solid lines indicate drug-protein interactions, color-coded by interaction type: *Targets* (therapeutic binding sites), *Enzymes* (metabolic conversion), *Carriers* (modifying pharmacokinetics) and *Transporters* (facilitating drug movement across membranes). Dotted lines show drug-indication relationships, color-coded by the highest clinical trial phase of the underlying association studies (Figure 4A). Linked pharmacogenomic information annotated between protein variants (e.g. SNP, haplotype) and a phenotype (e.g. efficacy, toxicity, dosing) can be retrieved from the ‘Network pharmacogenomics’ tab (Figure 4B).

This dynamic network display enables researchers to interactively dissect the molecular underpinnings of drug response variability (see use case examples), particularly in the context of on- and off-target effects. Users can conduct in-depth analyses of ATC-level interactions and identify patterns of protein variants (e.g. SNPs, haplotypes) that may influence phenotypic outcomes like efficacy, toxicity, or dosing thereby supporting the rationalization of drug response variability.

### Use case 1—exploring the relationship between molecular interaction profiles and pharmacogenomics

*Objective:* Find the connection between drug response variability, protein interaction, and genetic association for a drug of interest (here warfarin).

*How:* We first search for warfarin in ‘Drug search’ to obtain its ATC code (B01AA03). Next, we navigate to B01AA03 and choose the ‘Network pharmacogenomics’ tab.

*Result:* On the same page, we see clinical PGx data, the pharmacodynamic or pharmacokinetic relationships alongside the primary evidence for drug-response variability. Interestingly, when navigating to ‘gene-based burden data’, we can see that several proteins in the network also show a drug-associated burden association for different aggregates of population mutations (e.g, pLoFs for warfarin and ALB). Opening the gene-view for ALB offers a closer look at the various mutations, providing an opportunity to generate hypotheses for further investigation. For instance, an *in vitro* pharmacologist might select specific mutations based on the 3D protein structure and/or those with strong variant effect scores, which could form the basis for experimental testing to determine which mutations drive the cellular effects of warfarin-mediated drug-response variability.

### Use case 2—comparing drug adverse reaction profiles of two similar ATC codes

*Objective:* Compare the adverse reactions of drugs in similar ATC codes. Here, we compare adverse reactions of drugs in ‘Non-selective monoamine reuptake inhibitors’ (ATC: N06AA) with those from ‘Selective serotonin reuptake inhibitors’ (ATC: N06AB). Both Anticholinergic agents and Dopaminergic agents fall under ‘Antidepressants’ (ATC: N06A).

*How:* Navigate to N06AA and select ‘Compare network adverse drug reactions’ in ‘Network comparison’ tab and add N06AB as a comparator.

*Result:* We note that N06AB contains fewer drugs with fewer drug-protein interactions (234 versus 86 respectively), while displaying more drug-indication associations (74 versus 133). The result table lists all available ADRs for each drug, together with the frequency observed in the surveyed population. Noticeably, N06AB displays more ADRs for more drugs in the network, than N06AA, highlighting potential off-target ADRs given the polypharmacology of these agents, that dedicated drug discovery efforts might want to consider avoiding.

### Use case 3—evaluate the overall impact of a variant

*Objective*: Find the predicted impact of a protein-coding genetic variant. Given a healthcare professional identifies a variant of interest in a patient, which has not been listed on drug labels for PGx-testing previously.

*How*: Navigating to the ‘Variant search’ page, enter the variant identifier (e.g. 9_35 057 179_T/C) in the search box.

*Result*: An overview page showing basic information such as consequence, affected position in the encoded protein alongside other VEP algorithms, and a violin plot summarizing the scores are visible. A protein structural render highlights the position of the mutation after clicking on the corresponding amino acid position (in this case a start-lost mutation at position one). It's an ultra-rare mutation, with some VEP’s (e.g. BayesDel) predicting a strong effect, which is not surprising given the likely faulty expression of the protein. After selecting ‘Hide VEP scores’, we can navigate to ‘Show variant's associate statistics’, which displays some health phenotype associations for the selected variant, which the health professional might consider for cross-diagnosis and patient anamnesis.

## Discussion

Despite the importance of pharmacogenomics as a major contributor to drug response variability, only 120 drug-gene pairs are listed at the US Food and Drug Administration and only 70 at the European Medicines Agency that require genetic testing (38) (https://www.fda.gov/drugs/science-and-research-drugs/table-pharmacogenomic-biomarkers-drug-labeling). Most variants in drug-interacting proteins are not described in the literature or have not systematically been linked to adverse effects, which are estimated to be underreported by 90–99% (39,40). Additionally, most variants are rare or have minor consequences, and drug effects are confounded by general patient characteristics, co-medications, lifestyle factors, and comorbidities (41,42). Hence, disentangling the contribution of gene variability requires integration across biological domains and disciplines enabling the generation of new hypotheses, which can be probed and cross-validated by molecular scientists or clinicians alike.

To facilitate this, we have developed PGxDB (https://pgx-db.org/) as a user-friendly, integrative platform that helps experimental researchers, data scientists, and clinicians navigate the complex relationships between genetic profiles, molecular interactions, and therapeutic responses. To this end, we have generated >6,000 indication-drug-protein interaction networks covering all ATC levels (Supplementary Figure S1) and made these data available to the public both via custom downloads and programmatic interfaces (Figure 3H). PGxDB enables the comparison and contrast of molecular target and adverse reaction profiles across drugs from different therapeutic and chemical groups, a novel feature that sets it apart from other tools.

The data analysis and visualization tools of PGxDB enable researchers and healthcare professionals to easily navigate complex genetic information and understand the intricate relationships between genetic variability and clinical drug responses from different populations without consulting multiple databases. We explore aspects of this resource relating to PGx associations and genetic profiles, and we highlight some examples and potential use cases. This exploration raises important questions, such as whether targets that are more frequently mutated are more likely to be associated with adverse drug reactions, or whether pleiotropic targets—those involved in multiple biological systems—are more often linked to ADRs. We hope this platform enables future work to fully assess the contribution of genetic variability and drug-target interactions towards the increasing burden of adverse drug reactions and high clinical failure rate (43).

PGxDB has some limitations, particularly in the consistency and coverage of ID-mappings integrating ADRs from SIDER (31). SIDER uses STITCH V4 (44) identifiers to represent drugs, which correspond to PubChem IDs after removing the prefixes (e.g. STITCH ID ‘CID100000085’ becomes PubChem ID ‘85’). Once converted to PubChem IDs, we used the UniChem API to map these PubChem IDs to DrugBank IDs, which did not return mappings for ∼30% of drugs. Although we considered using drug names to match entries between PGxDB and SIDER, we were cautious about this approach due to the potential for errors in name-based matching. Future versions of PGxDB might incorporate additional sources of ADRs such as the FDA Adverse Event Reporting System (FAERS) (https://open.fda.gov/data/faers/)), adverse drug events extracted from drug labels (45), and Polygenic risk scores for treatment choices (46). However, these datasets are not linked to compound identifiers, requiring named entity recognitions or extensive manual curation. Additionally, there is potential to include capabilities for the search and retrieval of variant-specific information from relevant studies directly from the primary literature (47). Another area of improvement could be the integration of bioactivity data (48) such as drug efficacy, potency and selectivity information, helping to better understand the molecular mechanisms behind drug responses and adverse effects. Finally, integrating drug-indications from the RepurposeDrugs (22) database is limited to the non-complete mappings provided by UniChem DrugBank IDs into standard InChIKeys, which we hope will be addressed in future iterations of UniChem.

Although ATC classification is widely used and serves as a global standard overseen by the WHO (49), there are several shortcomings. First, ATC does not always keep pace with the rapid development of new drugs and treatments and several drugs do not even have an ATC code. The ATC system classifies drugs based on their therapeutic use, but it does not provide information about dosages or formulation strengths, which are crucial in clinical practice. For instance, a single ATC code may cover different dosages that could have very different clinical effects. The classification of combination drugs, i.e. those with more than one active ingredient, can be inconsistent or incomplete, as the system has to choose one main therapeutic group for the combination, even if both active ingredients serve different purposes. However, despite these limitations, ATC classification remains valuable for comparing grouped drugs in a network-based approach, as it provides a structured way to categorize drugs by their therapeutic use, allowing for meaningful insights into drug interactions and similarities, especially when analyzing broad patterns across multiple drug classes.

Looking forward, PGxDB has the potential to bridge the gap between molecular omics integration and clinical decision-making towards a more efficient genetically tailored drug discovery and drug development pipeline. We hope this tool will facilitate the discovery of new drug-gene-variant interactions and we encourage the community to further modify and extend the current implementation and potential application areas towards a deeper understanding of pharmacogenetic mechanisms. Finally, PGxDB could serve as an educational tool for students and professionals new to the field, promoting a wider understanding and adoption of pharmacogenomic principles.

## Supplementary Material

gkae1127_Supplemental_File

## Data Availability

All data is available via the web and GitHub (https://github.com/Duong-NguyenTrinhTrung/pgx-db). Documentation and tutorials are available at https://pgx-documentation.readthedocs.io. All open-source code can be obtained from figshare (10.6084/m9.figshare.26538574) or GitHub under the permissive Apache 2.0 License (https://www.apache.org/licenses/LICENSE-2.0). In addition, our database aggregates data from various publicly available database sources. To ensure transparency, we provide information (Supplementary Table S6) listing each source with its corresponding license.

## References

[1] Relling M.V. , Evans W.E. Pharmacogenomics in the clinic. Nature. 2015; 526:343–350. 26469045 10.1038/nature15817 PMC4711261

[2] Phillips K.A. , Veenstra D.L., Oren E., Lee J.K., Sadee W. Potential role of pharmacogenomics in reducing adverse drug reactions: a systematic review. JAMA. 2001; 286:2270–2279. 11710893 10.1001/jama.286.18.2270

[3] Lauschke V.M. , Zhou Y., Ingelman-Sundberg M. Pharmacogenomics beyond single common genetic variants: the way forward. Annu. Rev. Pharmacol. Toxicol. 2024; 64:33–51. 37506333 10.1146/annurev-pharmtox-051921-091209

[4] Swen J.J. , van der Wouden C.H., Manson L.E., Abdullah-Koolmees H., Blagec K., Blagus T., Böhringer S., Cambon-Thomsen A., Cecchin E., Cheung K.-C. A 12-gene pharmacogenetic panel to prevent adverse drug reactions: an open-label, multicentre, controlled, cluster-randomised crossover implementation study. Lancet North Am. Ed. 2023; 401:347–356. 10.1016/S0140-6736(22)01841-4 36739136

[5] Patel J.N. Cancer pharmacogenomics, challenges in implementation, and patient-focused perspectives. Pharmacogenom. Personal. Med. 2016; 9:65–77. 10.2147/PGPM.S62918 PMC4948716 27471406

[6] Pirmohamed M. Personalized pharmacogenomics: predicting efficacy and adverse drug reactions. Annu. Rev. Genomics Hum. Genet. 2014; 15:349–370. 24898040 10.1146/annurev-genom-090413-025419

[7] Pirmohamed M. Pharmacogenomics: current status and future perspectives. Nat. Rev. Genet. 2023; 6:350–362. 10.1038/s41576-022-00572-8 36707729

[8] Kizilkaya H.S. , Sørensen K.V., Madsen J.S., Lindquist P., Douros J.D., Bork-Jensen J., Berghella A., Gerlach P.A., Gasbjerg L.S., Mokrosiński J. et al. Characterization of genetic variants of GIPR reveals a contribution of β-arrestin to metabolic phenotypes. Nat. Metab. 2024; 6:1268–1281. 38871982 10.1038/s42255-024-01061-4 PMC11272584

[9] Burt T. , Dhillon S. Pharmacogenomics in early-phase clinical development. Pharmacogenomics. 2013; 14:1085–1097. 23837482 10.2217/pgs.13.81 PMC4551460

[10] Whirl-Carrillo M. , Huddart R., Gong L., Sangkuhl K., Thorn C.F., Whaley R., Klein T.E. An evidence-based framework for evaluating pharmacogenomics knowledge for personalized medicine. Clin. Pharmacol. Ther. 2021; 110:563–572. 34216021 10.1002/cpt.2350 PMC8457105

[11] Sim S.C. , Ingelman-Sundberg M. The Human Cytochrome P450 (CYP) Allele Nomenclature website: a peer-reviewed database of CYP variants and their associated effects. Hum. Genomics. 2010; 4:1–4. 10.1186/1479-7364-4-4-278 PMC3525213 20511141

[12] Relling M. , Klein T. CPIC: clinical pharmacogenetics implementation consortium of the pharmacogenomics research network. Clin. Pharmacol. Ther. 2011; 89:464–467. 21270786 10.1038/clpt.2010.279 PMC3098762

[13] Sangkuhl K. , Whirl-Carrillo M., Whaley R.M., Woon M., Lavertu A., Altman R.B., Carter L., Verma A., Ritchie M.D., Klein T.E. Pharmacogenomics clinical annotation tool (Pharm CAT). Clin. Pharmacol. Ther. 2020; 107:203–210. 31306493 10.1002/cpt.1568 PMC6977333

[14] Rubinstein W.S. , Maglott D.R., Lee J.M., Kattman B.L., Malheiro A.J., Ovetsky M., Hem V., Gorelenkov V., Song G., Wallin C. The NIH genetic testing registry: a new, centralized database of genetic tests to enable access to comprehensive information and improve transparency. Nucleic Acids Res. 2012; 41:D925–D935. 23193275 10.1093/nar/gks1173 PMC3531155

[15] Landrum M.J. , Lee J.M., Riley G.R., Jang W., Rubinstein W.S., Church D.M., Maglott D.R. ClinVar: public archive of relationships among sequence variation and human phenotype. Nucleic Acids Res. 2014; 42:D980–D985. 24234437 10.1093/nar/gkt1113 PMC3965032

[16] Yu Y. , Wang Y., Xia Z., Zhang X., Jin K., Yang J., Ren L., Zhou Z., Yu D., Qing T. PreMedKB: an integrated precision medicine knowledgebase for interpreting relationships between diseases, genes, variants and drugs. Nucleic Acids Res. 2019; 47:D1090–D1101. 30407536 10.1093/nar/gky1042 PMC6324052

[17] Musa A. , Tripathi S., Dehmer M., Yli-Harja O., Kauffman S.A., Emmert-Streib F. Systems pharmacogenomic landscape of drug similarities from LINCS data: drug Association Networks. Sci. Rep. 2019; 9:7849. 31127155 10.1038/s41598-019-44291-3 PMC6534546

[18] Knox C. , Wilson M., Klinger C.M., Franklin M., Oler E., Wilson A., Pon A., Cox J., Chin N.E., Strawbridge S.A. DrugBank 6.0: the DrugBank knowledgebase for 2024. Nucleic Acids Res. 2024; 52:D1265–D1275. 37953279 10.1093/nar/gkad976 PMC10767804

[19] Zdrazil B. , Felix E., Hunter F., Manners E.J., Blackshaw J., Corbett S., de Veij M., Ioannidis H., Lopez D.M., Mosquera J.F. The ChEMBL Database in 2023: a drug discovery platform spanning multiple bioactivity data types and time periods. Nucleic Acids Res. 2024; 52:D1180–D1192. 37933841 10.1093/nar/gkad1004 PMC10767899

[20] The UniProt Consortium UniProt: the universal protein knowledgebase in 2021. Nucleic Acids Res. 2020; 49:D480–D489. 10.1093/nar/gkaa1100 PMC7778908 33237286

[21] Jumper J. , Evans R., Pritzel A., Green T., Figurnov M., Ronneberger O., Tunyasuvunakool K., Bates R., Žídek A., Potapenko A. Highly accurate protein structure prediction with AlphaFold. Nature. 2021; 596:583–589. 34265844 10.1038/s41586-021-03819-2 PMC8371605

[22] Ianevski A. , Kushnir A., Nader K., Miihkinen M., Xhaard H., Aittokallio T., Tanoli Z. RepurposeDrugs: an interactive web-portal and predictive platform for repurposing mono- and combination therapies. Brief. Bioinf. 2024; 25:bbae328. 10.1093/bib/bbae328 PMC11232279 38980370

[23] Piñero J. , Bravo À., Queralt-Rosinach N., Gutiérrez-Sacristán A., Deu-Pons J., Centeno E., García-García J., Sanz F., Furlong L.I. DisGeNET: a comprehensive platform integrating information on human disease-associated genes and variants. Nucleic Acids Res. 2016; 45:833–839. 10.1093/nar/gkw943 PMC5210640 27924018

[24] Chambers J. , Davies M., Gaulton A., Hersey A., Velankar S., Petryszak R., Hastings J., Bellis L., McGlinchey S., Overington J.P. UniChem: a unified chemical structure cross-referencing and identifier tracking system. J. Cheminform. 2013; 5:3. 23317286 10.1186/1758-2946-5-3 PMC3616875

[25] Malone J. , Holloway E., Adamusiak T., Kapushesky M., Zheng J., Kolesnikov N., Zhukova A., Brazma A., Parkinson H. Modeling sample variables with an Experimental Factor Ontology. Bioinformatics. 2010; 26:1112–1118. 20200009 10.1093/bioinformatics/btq099 PMC2853691

[26] Lipscomb C.E. Medical subject headings (MeSH). Bull. Med. Libr. Assoc. 2000; 88:265. 10928714 PMC35238

[27] Karczewski K.J. , Solomonson M., Chao K.R., Goodrich J.K., Tiao G., Lu W., Riley-Gillis B.M., Tsai E.A., Kim H.I., Zheng X. Systematic single-variant and gene-based association testing of thousands of phenotypes in 394,841 UK Biobank exomes. Cell Genomics. 2022; 2:100168. 36778668 10.1016/j.xgen.2022.100168 PMC9903662

[28] McLaren W. , Gil L., Hunt S.E., Riat H.S., Ritchie G.R., Thormann A., Flicek P., Cunningham F. The ensembl variant effect predictor. Genome Biol. 2016; 17:1–14. 27268795 10.1186/s13059-016-0974-4 PMC4893825

[29] Liu X. , Li C., Mou C., Dong Y., Tu Y. dbNSFP v4: a comprehensive database of transcript-specific functional predictions and annotations for human nonsynonymous and splice-site SNVs. Genome Med. 2020; 12:1–8. 10.1186/s13073-020-00803-9 PMC7709417 33261662

[30] Cheng J. , Novati G., Pan J., Bycroft C., Žemgulytė A., Applebaum T., Pritzel A., Wong L.H., Zielinski M., Sargeant T. Accurate proteome-wide missense variant effect prediction with AlphaMissense. Science. 2023; 381:eadg7492. 37733863 10.1126/science.adg7492

[31] Kuhn M. , Letunic I., Jensen L.J., Bork P. The SIDER database of drugs and side effects. Nucleic Acids Res. 2016; 44:D1075–D1079. 26481350 10.1093/nar/gkv1075 PMC4702794

[32] Große-Michaelis I. , Proestel S., Rao R.M., Dillman B.S., Bader-Weder S., Macdonald L., Gregory W. MedDRA Labeling Groupings to Improve Safety Communication in Product Labels. Ther. Innov. Regul. Sci. 2023; 57:1–6. 35939205 10.1007/s43441-022-00393-1 PMC9810671

[33] McKinney W. pandas: a foundational Python library for data analysis and statistics. Python High Perform. Sci. Comput. 2011; 14:1–9.

[34] Harris C.R. , Millman K.J., Van Der Walt S.J., Gommers R., Virtanen P., Cournapeau D., Wieser E., Taylor J., Berg S., Smith N.J. Array programming with NumPy. Nature. 2020; 585:357–362. 32939066 10.1038/s41586-020-2649-2 PMC7759461

[35] Thompson M.D. , Reiner-Link D., Berghella A., Rana B.K., Rovati G.E., Capra V., Gorvin C.M., Hauser A.S. G protein-coupled receptor (GPCR) pharmacogenomics. Crit. Rev. Clin. Lab. Sci. 2024; 1–44. 10.1080/10408363.2024.2358304 39119983

[36] Viacava Follis A. Centrality of drug targets in protein networks. BMC Bioinf. 2021; 22:1–29. 10.1186/s12859-021-04342-x PMC8555226 34715787

[37] Badkas A. , De Landtsheer S., Sauter T. Topological network measures for drug repositioning. Brief. Bioinf. 2021; 22:bbaa357. 10.1093/bib/bbaa357 PMC8294518 33348366

[38] Shekhani R. , Steinacher L., Swen J.J., Ingelman-Sundberg M. Evaluation of current regulation and guidelines of pharmacogenomic drug labels: opportunities for improvements. Clin. Pharmacol. Ther. 2020; 107:1240–1255. 31715018 10.1002/cpt.1720 PMC7232863

[39] Hazell L. , Shakir S.A. Under-reporting of adverse drug reactions. Drug Saf. 2006; 29:385–396. 16689555 10.2165/00002018-200629050-00003

[40] Giacomini K.M. , Krauss R.M., Roden D.M., Eichelbaum M., Hayden M.R., Nakamura Y. When good drugs go bad. Nature. 2007; 446:975–977. 17460642 10.1038/446975a

[41] Bomba L. , Walter K., Soranzo N. The impact of rare and low-frequency genetic variants in common disease. Genome Biol. 2017; 18:1–17. 28449691 10.1186/s13059-017-1212-4 PMC5408830

[42] Moc C. Pharmacogenomics: an evolving clinical tool for precision medicine. Cleve. Clin. J. Med. 2020; 87:91. 32015062 10.3949/ccjm.87a.19073

[43] Meyer U.A. Pharmacogenetics and adverse drug reactions. Lancet North Am. Ed. 2000; 356:1667–1671. 10.1016/S0140-6736(00)03167-6 11089838

[44] Kuhn M. , Szklarczyk D., Pletscher-Frankild S., Blicher T.H., von Mering C., Jensen L.J., Bork P. STITCH 4: integration of protein–chemical interactions with user data. Nucleic Acids Res. 2013; 42:D401–D407. 24293645 10.1093/nar/gkt1207 PMC3964996

[45] Tanaka Y. , Chen H.Y., Belloni P., Gisladottir U., Kefeli J., Patterson J., Srinivasan A., Zeitz M., Sirdeshmukh G., Berkowitz J. OnSIDES (ON-label SIDE effectS resource) database: extracting Adverse Drug Events from Drug Labels using Natural Language Processing Models. 2024; Medrxiv doi:24 March 2024, pre-print: not peer-reviewed https://www.medrxiv.org/content/10.1101/2024.03.22.24304724v1. 10.1016/j.medj.2025.100642 PMC12256195 40179876

[46] Lewis C.M. , Vassos E. Polygenic risk scores: from research tools to clinical instruments. Genome Medicine. 2020; 12:44. 32423490 10.1186/s13073-020-00742-5 PMC7236300

[47] Allot A. , Wei C.-H., Phan L., Hefferon T., Landrum M., Rehm H.L., Lu Z. Tracking genetic variants in the biomedical literature using LitVar 2.0. Nat. Genet. 2023; 55:901–903. 37268776 10.1038/s41588-023-01414-x PMC11096795

[48] Zdrazil B. , Felix E., Hunter F., Manners E.J., Blackshaw J., Corbett S., de Veij M., Ioannidis H., Lopez D.M., Mosquera J.F. et al. The ChEMBL Database in 2023: a drug discovery platform spanning multiple bioactivity data types and time periods. Nucleic Acids Res. 2023; 52:D1180–D1192. 10.1093/nar/gkad1004 PMC10767899 37933841

[49] Hollingworth S. , Kairuz T. Measuring medicine use: applying ATC/DDD methodology to real-world data. pharm. 2021; 9:60. 10.3390/pharmacy9010060 PMC8006033 33802774

