# Supplementary material for: PGxDB: an interactive web-platform for pharmacogenomics research
Source: Nucleic Acids Res. 2024 Nov 20;53(D1):D1486–97. doi: 10.1093/nar/gkae1127 (PMC11701576; doi:10.1093/nar/gkae1127)
Supplement: gkae1127_Supplemental_File [file gkae1127_supplemental_file.pdf]

SUPPLEMENTARY DATA

PGxDB: An interactive web-platform for pharmacogenomics research

Trinh Trung Duong Nguyen[1], Ziaurrehman Tanoli[2,3], Saad Hassan[3], Umut Onur Özcan[2], Jimmy Caroli[1], Albert J. Kooistra[1], David E. Gloriam[1], Alexander S. Hauser[1]*

[1]Department of Drug Design and Pharmacology, Faculty of Health and Medical Sciences, University of Copenhagen, 2100 Copenhagen, Denmark.
[2]Institute for Molecular Medicine Finland (FIMM), HiLIFE, University of Helsinki, Finland,
[3]BioICAWtech, Helsinki, Finland
* To whom correspondence should be addressed. Email: alexander.hauser@sund.ku.dk

URL: https://pgx-db.org/ or https://pgxdb.org/
Keywords: pharmacogenomics, disease-variant associations, ATC, drug-target-network

Table S1: Comparison of PGxDB and other relevant resources

**Feature**	**PGxDB**	**PharmGKB** [10]	**ClinVar** [15]	**CYP** [11] (*)	**PharmCAT** [13]	**GTR** [14]	**PreMedKB** [16]	**Gene-bass** [18]	**Ensembl and plug-ins** [19-21] (**)	**LINCS DANs (Drug assocation networks)** [17] (*)
Data on variant affecting drug responses	✓	✓	✓	✓ Metabolizing enzymes only	✓	✓	✓	✓	✗	✗
REST APIs For programmable access	✓	✗	✗	✗	✗	✗	✗	✗	✓	✗
Indication-drug-gene network visualization and analysis tools	✓	✗	✗	✗	✗	✗	✓	✗	✗	✓ Only drug-drug
Combines drug profiles indexed at the ATC level with genetic data	✓	✗	✗	✗	✗	✗	✗	✗	✗	✓ Drug similarities

Interlinks data on drugs and genes	✓	✓	✓	✓	✓	✗	✓	✓	✗	✗
Interlinks data on drugs and adverse reactions	✓	✓	✗	✗	✗	✗	✗	✗	✗	✗
Interlinks data on genes and indications	✓	✓	✓	✓	✗	✓	✓	✓	✗	✗
Variant effect prediction and visualization (***)	✓	✗	✗	✗	✗	✗	✗	✗	✓ No visual-ization	✗

(*): the website is not accessible at the moment
(**): for variant effect prediction scores
(***): from 41 variant prediction algorithms

PGxDB stands out from other resources by offering a comprehensive range of features that are not collectively available elsewhere. It integrates data on variant-drug responses, with full variant effect predictions from 41 algorithms and variant association study statistics to give users a comprehensive view of variants. It also uniquely combines drug profiles grouped by therapeutic, pharmacological, and chemical properties (ATC codes) with genetic data, and interlinks drugs, genes, and indications within a single platform. Additionally, PGxDB visualizes and analyzes drug-gene-indication network topologies and offers tools for exploring adverse drug reactions. PGxDB also supports REST APIs for programmable access, allowing seamless integration into custom workflows. These features differentiate PGxDB from other platforms, such as PharmGKB, ClinVar, Genebass, and others, which either lack certain types of integration (e.g., ATC-level drug indexing or variant effect predictions) or provide limited data access and visualization capabilities.

Table S2: Selected phenotype association categories from the UK Biobank.

No.	Phenotype category
1	Health-related outcomes > First occurrences > Pregnancy, childbirth and the puerperium
2	Health-related outcomes > First occurrences > Eye and adnexa disorders
3	Health-related outcomes > First occurrences > Respiratory system disorders
4	Health-related outcomes > First occurrences > Musculoskeletal system and connective tissue disorders
5	Health-related outcomes > First occurrences > Genitourinary system disorders
6	Health-related outcomes > First occurrences > Mental and behavioural disorders
7	Health-related outcomes > First occurrences > Digestive system disorders
8	Health-related outcomes > First occurrences > Ear and mastoid process disorders
9	Health-related outcomes > First occurrences > Circulatory system disorders
10	Health-related outcomes > First occurrences > Nervous system disorders

11	Health-related outcomes > First occurrences on> Skin and subcutaneous tissue disorders
12	Health-related outcomes > First occurrences > Certain infectious and parasitic diseases
13	Health-related outcomes > First occurrences > Blood, blood-forming organs and certain immune disorders
14	Health-related outcomes > First occurrences > Endocrine, nutritional and metabolic diseases"
15	Health-related outcomes > First occurrences > Congenital disruptions and chromosomal abnormalities
16	Health-related outcomes > First occurrences > Certain conditions originating in the perinatal period
17	UK Biobank Assessment Centre > Verbal interview > Medications

Table S3: List of included variant effect prediction algorithms and conservation scores. Users are encouraged to refer to the documentation of dbNSFP, version 4.2a, for full information

Variant effect prediction algorithm	Short description	Score range
Alpha Missense Pathogenicity	Pathogenicity prediction scores output from a state-of-the-art deep learning model	[0,1]**
BayesDel (addAF) rankscore	*BayesDel (addAF) rankscore* were ranked among all BayesDel (addAF) scores in dbNSFP.	[0,1]
BayesDel (noAF) rankscore	*BayesDel (noAF) rankscores* were ranked among all BayesDel (noAF) scores in dbNSFP.	[0,1]
bStatistic converted rankscore	Data from CADD v1.4. bStatistic scores were converted to -bStatistic, then ranked among all -bStatistic scores in dbNSFP.	[0,1]
CADD raw rankscore (*)	*CADD raw rankscores* were ranked among all CADD raw scores in dbNSFP.	[0,1]
ClinPred rankscore	*ClinPred rankscores* were ranked among all ClinPred scores in dbNSFP.	[0,1]
DANN rankscore	*DANN rankscores* were ranked among all DANN scores in dbNSFP	[0,1]
DEOGEN2 rankscore	*DEOGEN2 rankscores* were ranked among all DEOGEN2 scores in dbNSFP	[0,1]
Eigen_PC raw coding rankscore	*Eigen_PC raw coding rankscore*s were ranked among all Eigen-PC-raw scores in dbNSFP	[0,1]
Eigen raw coding rankscore	*Eigen-raw rankscores* were then ranked among all Eigen-raw scores in dbNSFP.	[0,1]
FATHMM_converted rankscore	*FATHMM_converted_rankscore*: FATHMMori scores were first converted to FATHMMnew=1-(FATHMMori+16.13)/26.77, then ranked among all FATHMMnew scores in dbNSFP.	[0,1]**
Fathmm_ MKL coding rankscore	*Fathmm-MKL_coding_score*: fathmm-MKL p-values. Scores range from 0 to 1. SNVs with scores >0.5 are predicted to be deleterious, and those <0.5 are predicted	[0,1]

	to be neutral or benign. Scores close to 0 or 1 are with the highest-confidence. Coding scores are trained using 10 groups of features. More details of the score can be found in doi: 10.1093/bioinformatics/btv009. *Fathmm-MKL_coding_rankscore* were then ranked among all fathmm-MKL coding scores in dbNSFP.	
Fathmm_XF coding rankscore	*Fathmm-XF_coding rankscores* were then ranked among all fathmm-XF coding scores in dbNSFP.	[0,1]
GM12878_fitCons rankscore	*GM12878_fitCons rankscores* were then ranked among all GM12878 fitCons scores in dbNSFP.	[0,1]
Geno Canyon rankscore	*GenoCanyon rankscores* were ranked among all integrated fitCons scores in dbNSFP.	[0,1]
GERP++_RS rankscore	GERP++ RS scores were ranked among all GERP++ RS scores in dbNSFP.	[0,1]
H1_hESC_fitCons rankscore	*H1-hESC fitCons rankscores* were then ranked among all H1-hESC fitCons scores in dbNSFP.	[0,1]
HUVEC_fitCons rankscore	*HUVEC fitCons rankscores* were ranked among all HUVEC fitCons scores in dbNSFP.	[0,1]
Integrated_fitCons rankscore	I*ntegrated fitCons rankscores* were then ranked among all integrated fitCons scores in dbNSFP	[0,1]
LRT converted rankscore	*LRT converted rankscores*: The original LRT two-sided p-value (LRTori), ranges from 0 to 1.	[0.00162, 0.8433] **
LIST_S2 rankscore	*LIST-S2_rankscores*: LIST-S2 scores were ranked among all LIST-S2 scores in dbNSFP.	[0,1]
MetaLR rankscore	*MetaLR rankscores* were then ranked among all MetaLR scores in dbNSFP	[0,1]**
MetaRNN rankscore	MetaRNN rankscores were then ranked among all MetaRNN scores in dbNSFP	[0,1]**
MetaSVM rankscore	*MetaSVM rankscores* were then ranked among all MetaSVM scores in dbNSFP	[0,1]**
MutPred rankscore	*MutPred rankscores* were ranked among all *MutPred scores* in dbNSFP	[0,1]
MutationAssessor rankscore	*MutationAssessor rankscores*: MAori scores were ranked among all MAori scores in dbNSFP.	[0,1] **
MutationTaster converted rankscore	*MutationTaster scores*: MutationTaster p-value (MTori), ranges from 0 to 1. *MutationTaster converted rankscores*: The MTori scores were first converted. If the prediction is "A" or "D" MTnew=MTori; if the prediction is "N" or "P", MTnew=1-MTori. Then MTnew scores were ranked among all MTnew scores in dbNSFP. If there are multiple scores of a SNV, only the largest MTnew was used in ranking. The rankscore is the ratio of the rank of the score over the total number of MTnew scores in dbNSFP.	[0.08979, 0.81001] **
M_CAP rankscore	*M-CAP rankscores*: M-CAP scores were ranked among all M-CAP scores in dbNSFP	[0,1]
MPC rankscore	*MPC rankscores*: MPC scores were ranked among all MPC scores in dbNSFP	[0,1]

MVP rankscore	*MVP rankscores*: MVP scores were ranked among all MVP scores in dbNSFP.	[0,1]
Polyphen2_ HDIV rankscore	*Polyphen2 HDIV rankscores* were ranked among all *Polyphen2* HDIV scores in dbNSFP. The rankscore is the ratio of the rank the score over the total number of the scores in dbNSFP. If there are multiple scores, only the most damaging (largest) rankscore is presented.	[0.03061, 0.91137]**
Polyphen2_ HVAR rankscore	*Polyphen2 HVAR rankscores* were ranked among all Polyphen2 HVAR scores in dbNSFP. The rankscore is the ratio of the rank the score over the total number of the scores in dbNSFP. If there are multiple scores, only the most damaging (largest) rankscore is presented.	[0.01493, 0.97581]**
PROVEAN rankscore	PROVEAN score (PROVEANori). Scores range from -14 to 14. The smaller the score the more likely the SNP has damaging effect. *PROVEAN converted rankscores*: PROVEANori were first converted to PROVEANnew=1-(PROVEANori+14)/28, then ranked among all PROVEANnew scores in dbNSFP. The rankscore is the ratio of the rank the PROVEANnew score over the total number of PROVEANnew scores in dbNSFP. If there are multiple scores, only the most damaging (largest) rankscore is presented.	[0,1]
PhastCons 30way rankscore	*phastCons30way_mammalian rankscores*: *phastCons30way_mammalian scores* were ranked among all phastCons30way_mammalian scores in dbNSFP	[0,1]
Phylo_ P30way rankscore	*PhyloP30way_mammalian_rankscores*: phyloP30way_mammalian scores were ranked among all phyloP30way_mammalian scores in dbNSFP	[0,1]
PrimateAI rankscore	*PrimateAI rankscores*: PrimateAI scores were ranked among all PrimateAI scores in dbNSFP.	[0,1]
REVEL rankscore	*REVEL rankscores*: REVEL scores were ranked among all REVEL scores in dbNSFP.	[0,1]
SIFT4G converted rankscore	*SIFT4G converted rankscores*: SIFT4G scores were first converted to SIFT4Gnew=1-SIFT4G, then ranked among all SIFT4Gnew scores in dbNSFP. The rankscore is the ratio of the rank the SIFT4Gnew score over the total number of SIFT4Gnew scores in dbNSFP. If there are multiple scores, only the most damaging (largest) rankscore is presented.	[0,1]
SIFT rankscore	*SIFT_converted_rankscores*: SIFTori scores were first converted to SIFTnew=1-SIFTori, then ranked among all SIFTnew scores in dbNSFP. The rankscore is the ratio of the rank the SIFTnew score over the total number of SIFTnew scores in dbNSFP. If there are multiple scores, only the most damaging (largest) rankscore is presented.	[0.00964, 0.91255]**
SiPhy_29way_log Odds rankscore	*SiPhy_29way_logOdds rankscores*: SiPhy_29way_logOdds scores were ranked among all SiPhy_29way_logOdds scores in dbNSFP.	[0,1]
VEST4 rankscore (*)	*VEST4_rankscores*: VEST4 scores were ranked among all VEST4 scores in dbNSFP. The rankscore is the ratio	[0,1]

	of the rank of the score over the total number of VEST4 scores in dbNSFP. In case there are multiple scores for the same variant, the largest score (most damaging) is presented.	

(*): Commercial users should contact the appropriate authorities for further information.
(**): Ranges are explicitly stated in the corresponding studies compared to default understanding that the rank score is normally in the range of [0,1]

Table S4: Pages interlinked throughout PGxDB

Web icon	Entity	Menu	Browsing	Linked page	API
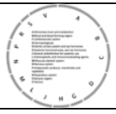	ATC code	ATC code	ATC classification browser	Itself	Yes
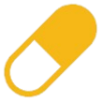	Drugs	Drugs	Drug search	ATC classification browser	Yes
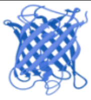	Targets	Targets	Target search	ATC classification browser	Yes
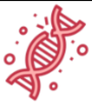	Variant	Variant	Variant search	Gene detail*	Yes
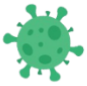	Indicati on	Indicati on	Indication search	ATC classification browser	Yes

(*): Gene detail page is a direct-access page that is not included in a menu

Table S5: ATC indication-drug-protein network comparison options.

Option	Function
Network size comparison	Comparing 2 networks based on number of nodes (drugs, targets and diseases), drug-target interactions, and drug-disease associations. This module can allow end users to detemine complexities between different networks.
Degree distribution comparison	Comparing 2 networks on distribution of degree of drug-disease association or drug-protein interaction nodes. This helps to understand the connectivity patterns between networks.
Mode of action distribution comparison	Examining the distribution of modes of action (target, transporter, enzyme) for drugs in both networks. This helps to identify if one network has a predominant mode of action.
Clinical trial phase distribution comparison	Analyzing the distribution of clinical trial phases for drug-disease associations in each network. This helps to understand the focus of clinical studies.

Degree of centraliztion comparison	Measuring the degree of centralization in each network to identify highly connected drug, disease or target nodes. This checks if one network has a more centralized structure than the other.
Average path length comparison	Calculating the average shortest path length between drug, disease or target nodes in each network. This compares the efficiency of information transfer within the networks.
Community comparison	Applying community detection algorithms to identify clusters or modules within each network. This compares the community structures to understand functional modules.
See common and unique network elements	Identifying the common drugs, targets, and diseases shared between the two networks. This highlights unique elements in each network to understand their specific characteristics.

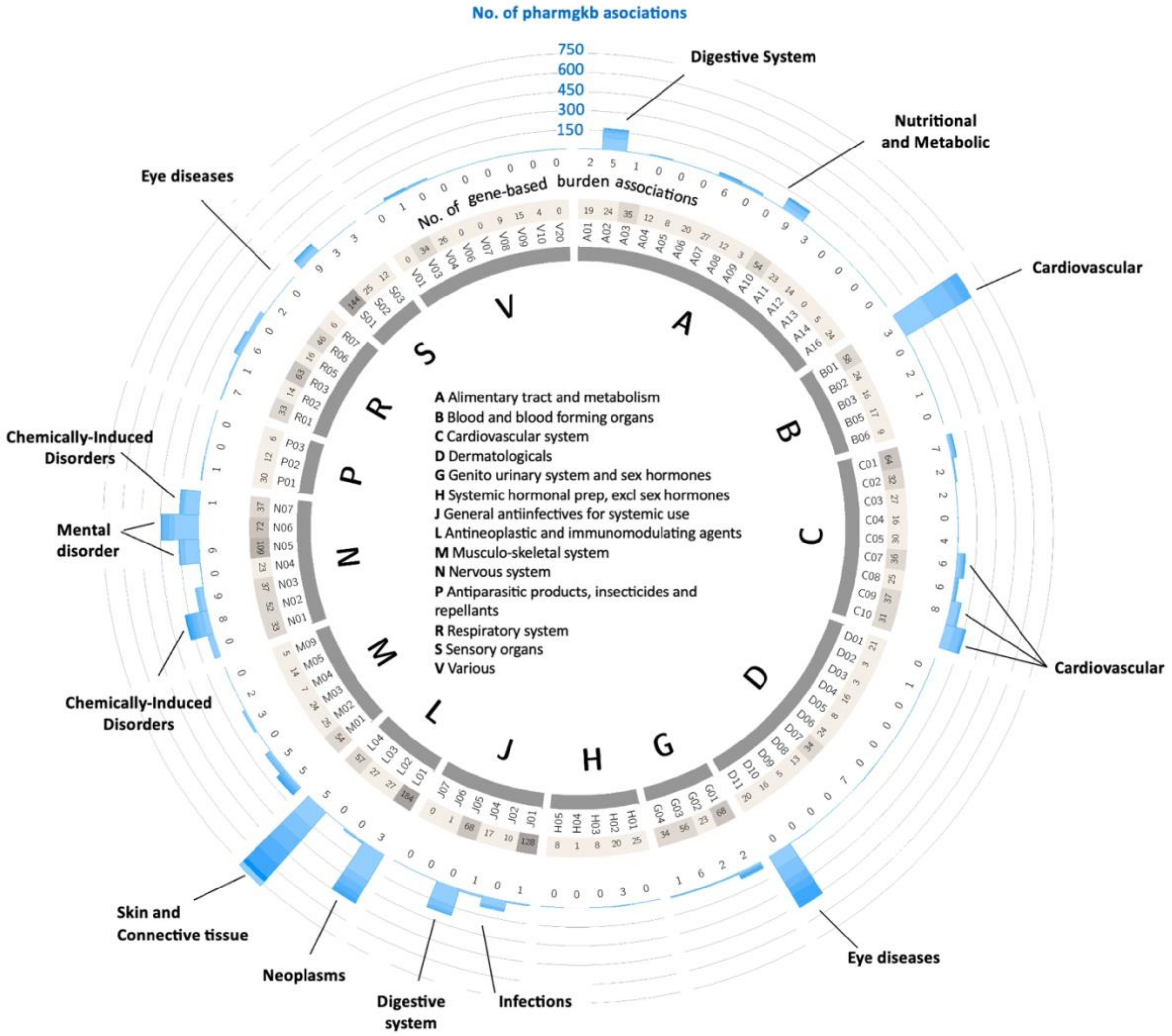

Figure S1: Distribution of drugs, annotated drug label associations, and burden test

associations in PGxDB across 14 anatomical categories from the Anatomical Therapeutic Chemical (ATC) classification. The ATC classification system classifies approved drugs with a seven-digit code divided into five levels. The second layer shows a histogram representing the count of drugs within each of the 93 therapeutic categories. The third layer displays the number of Genebass PGxDB associations for missense variants with a p-value less than 0.05. The outer-most layer displays the number of drug-label annotations from PharmGKB (filtered for significant findings below a p-value threshold of 0.05) as a stacked histogram representing 25 disease classes, with darker columns indicating a higher number of diseases in that class. The most frequent disease in each class is annotated by name. The figure was generated using Circos.

Table S6: Source databases and corresponding licenses

Database name	License type	License compatible with CC BY?	Notes
DrugBank	CC BY-NC 4.0	No	Non-commercial use only; restricts commercial redistribution
Genebass	CC BY 4.0	Yes	Also attribute to UK Biobank
PharmgKB	CC BY-SA 4.0	Partially	Requires derivative works to be shared under the same license
Ensembl	EMBL-EBI Terms of Use (General)	Yes	Free to use with attribution; additional terms may apply for specific data resources.
CIDER	CC BY-NC-SA 4.0	No	Non-commercial use only; derivative works must be shared under the same license.
ChEMBL	EMBL-EBI Terms of Use (General)	Yes	Free to use with attribution; additional terms may apply for specific data resources.
DisGeNET	Source database Licenses	Depend on source database licenses	DISGENET database incorporates data from 19 databases including UniProt
UniProt	CC BY 4.0	Yes	Free to use with attribution

Use case: "Compare the data extraction/analysis process of PGxDB with other databases using drugs from ATC code: L01BA"

We selected the Level 4 ATC code L01BA for this comparison. This level was chosen over Levels 1, 2, or 3 because it includes fewer drugs, which makes the analysis more manageable. We excluded Level 5 since it typically contains only a single drug. Using L01BA as our starting point, we compared PGxDB with other databases to demonstrate its efficiency for end users.

The following guide outlines a step-by-step process (accompanied by actual website snapshots) for extracting drug-targets and toxicities from DrugBank, adverse drug reactions from SIDER, and drug indications from RepurposeDrugs DB. We then repeated the same process using PGxDB and concluded with a time comparison for implementing this use case across PGxDB and other databases.

1. **Use DrugBank to retrieve information on the drugs, targets and toxicities.**

Accessing ATC Classification: On the DrugBank website, the ATC classification section is accessible from the top bar on the main page.

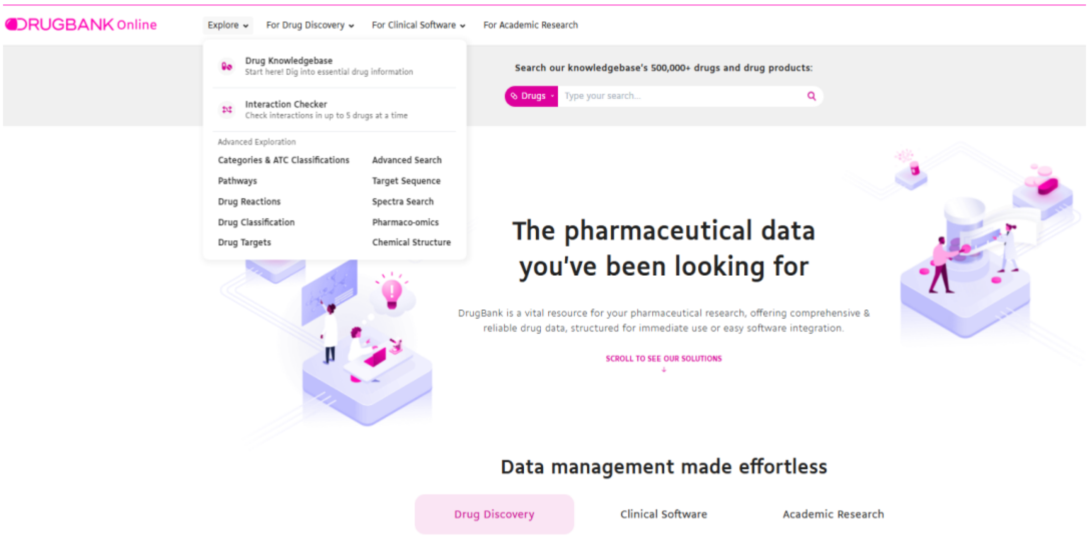

Navigating to ATC Classification: In the filter section, the "Browse ATC Classification" button is clicked to reach the ATC Classification search page.

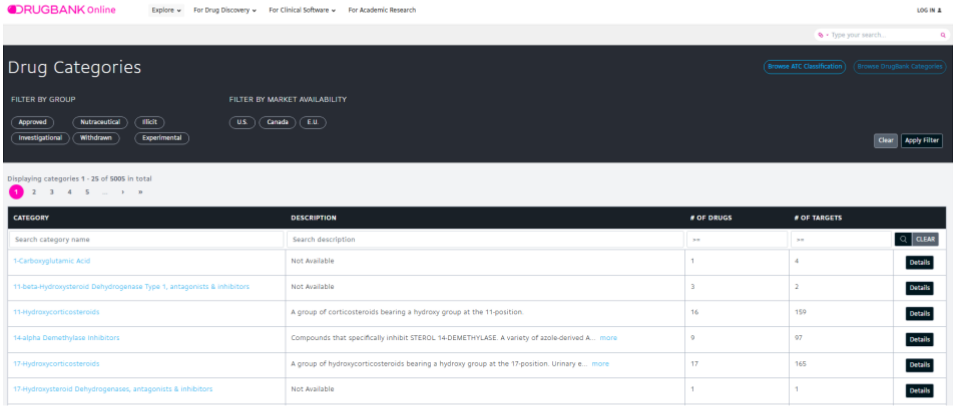

Searching for ATC Codes: In this example, we conducted two separate queries using the ATC codes L01BA and M04AA, which correspond to **"Folic Acid Analogues"** and **"Preparations Inhibiting Uric Acid Production"**, respectively. The L01BA code includes **7 drugs** and is associated with **76 drug targets** in total, as shown below.

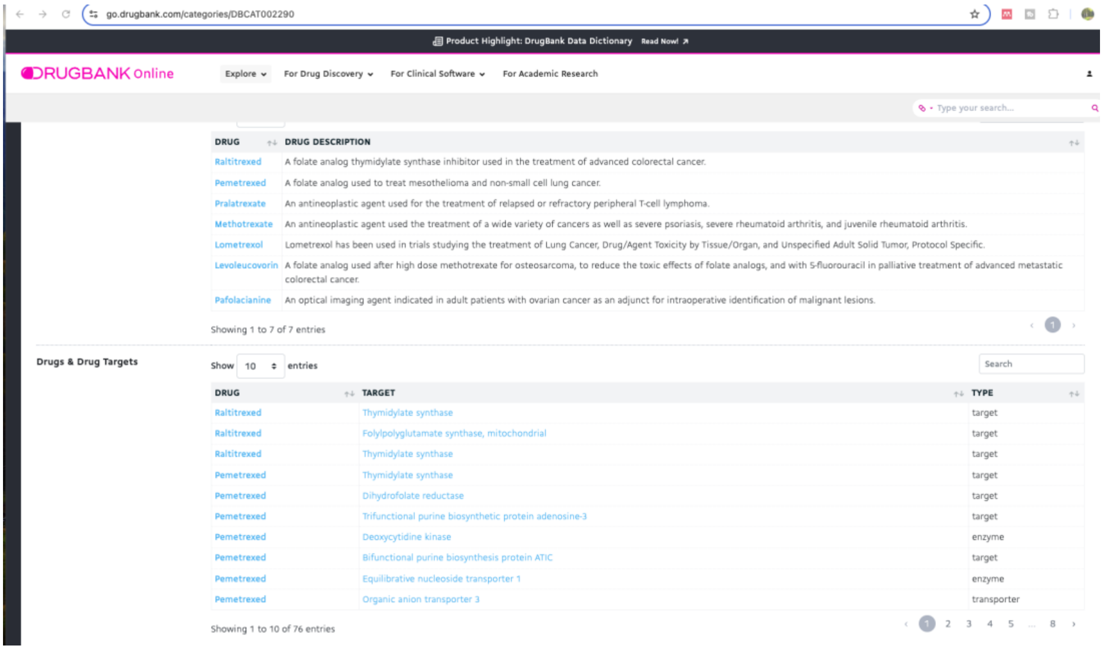

By opening the first drug ‘Raltitrexed’ in the new tab, the website hyperlinked us to the drugs page in the new tab, which shows toxicities and other drug information in detail as shown in the below snapshot.

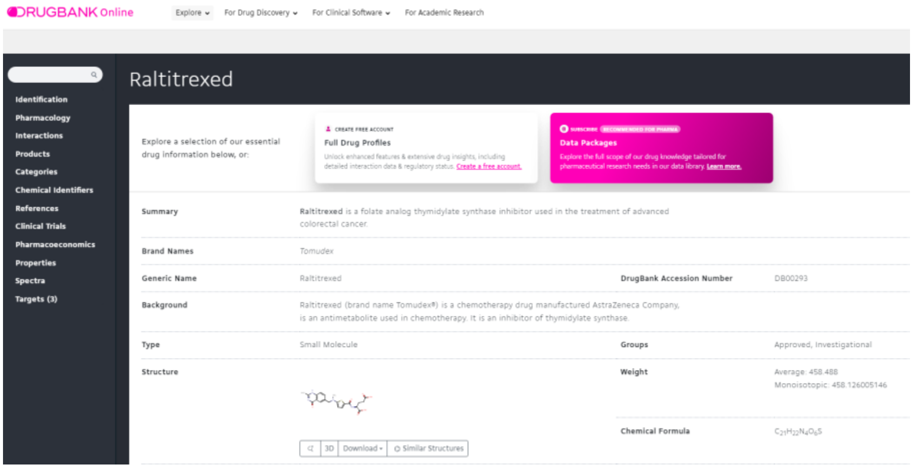

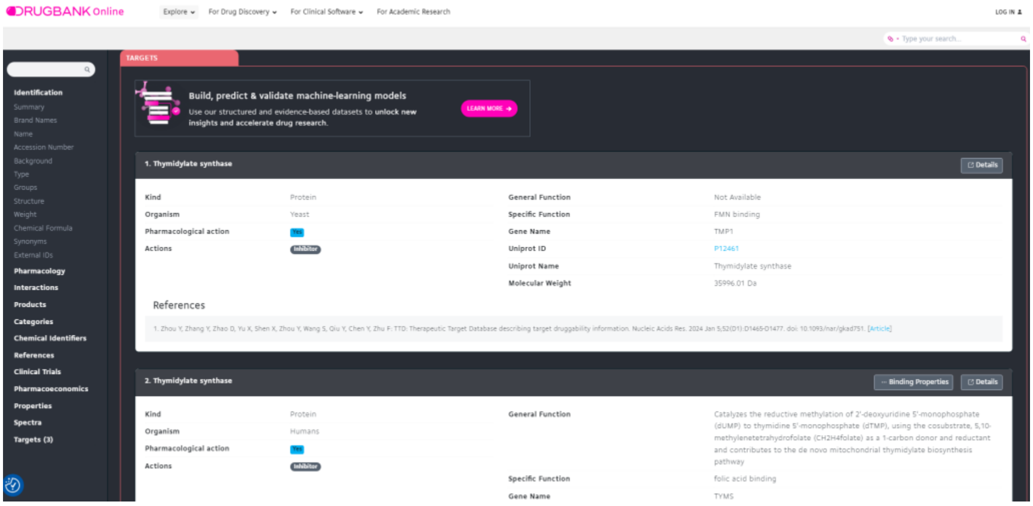

The user can find the toxicity section under Pharmacology title for the selected drugs as shown below.

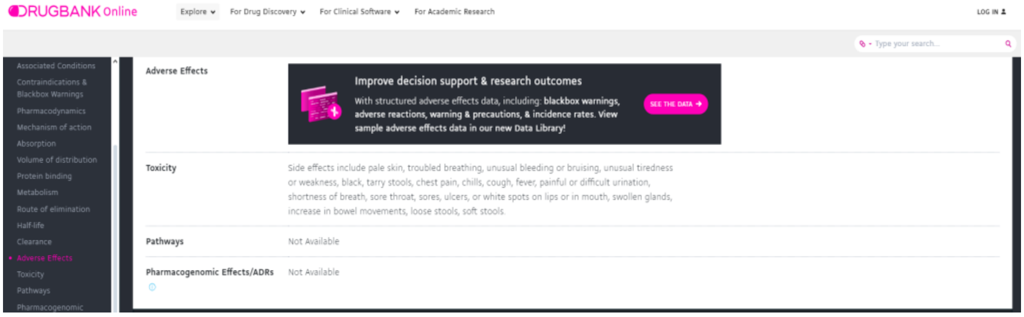

To check similar information for all drugs, you need to open those in new tabs and repeat the process.

2. **Next we used the SIDER database to retrieve ADRs associated with drugs belonging to ATC code: L01BA.**

Accessing SIDER: On the SIDER website, the ATC code search bar is found under the "Drug List" section.

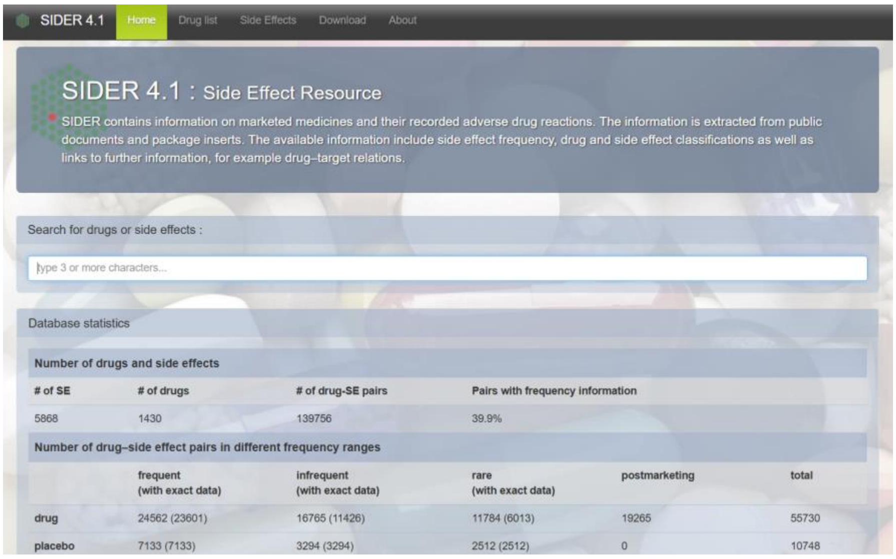

Search L01BA in SIDER DB: When we searched for the **L01BA** code in the SIDER database, only **four out of seven drugs** were found. However, SIDER does not provide an option to view adverse drug reactions (ADRs) for each drug in a separate tab. As a result, we had to spend additional time manually navigating through the correct ATC code, selecting each drug individually, and then analyzing its side effects or capturing snapshots. This repetitive process increased the time required for the analysis. Following the snapshots for this process using SIDER DB.

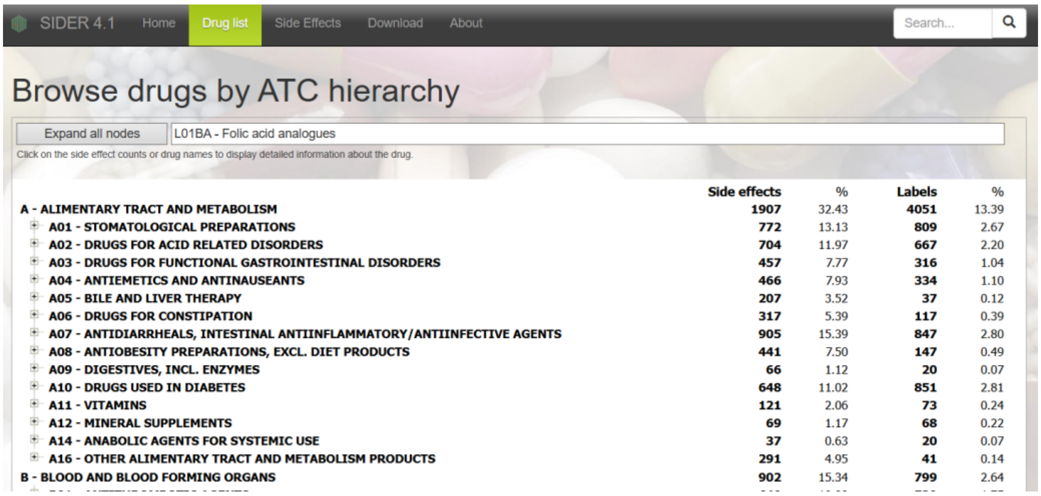

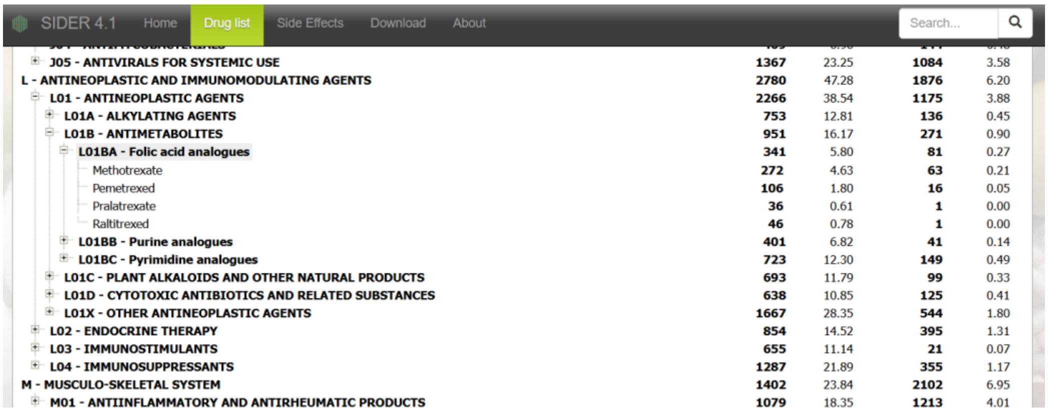

J05 - ANTIVIRALS FOR SYSTEMIC USE	1367	23.25	1084	3.58
L - ANTINEOPLASTIC AND IMMUNOMODULATING AGENTS	2780	47.28	1876	6.20
L01 - ANTINEOPLASTIC AGENTS	2266	38.54	1175	3.88
L01A - ALKYLATING AGENTS	753	12.81	136	0.45
L01B - ANTIMETABOLITES	951	16.17	271	0.90
L01BA - Folic acid analogues	341	5.80	81	0.27
Methotrexate	272	4.63	63	0.21
Pemetrexed	106	1.80	16	0.05
Pralatrexate	36	0.61	1	0.00
Raltitrexed	46	0.78	1	0.00
L01BB - Purine analogues	401	6.82	41	0.14
L01BC - Pyrimidine analogues	723	12.30	149	0.49
L01C - PLANT ALKALOIDS AND OTHER NATURAL PRODUCTS	693	11.79	99	0.33
L01D - CYTOTOXIC ANTIBIOTICS AND RELATED SUBSTANCES	638	10.85	125	0.41
L01X - OTHER ANTINEOPLASTIC AGENTS	1667	28.35	544	1.80
L02 - ENDOCRINE THERAPY	854	14.52	395	1.31
L03 - IMMUNOSTIMULANTS	655	11.14	21	0.07
L04 - IMMUNOSUPPRESSANTS	1287	21.89	355	1.17
M - MUSCULO-SKELETAL SYSTEM	1402	23.84	2102	6.95
M01 - ANTIINFLAMMATORY AND ANTIRHEUMATIC PRODUCTS	1079	18.35	1213	4.01

Reviewing ADRs: The following snapshot displays the adverse drug reactions (ADRs) table for **Raltitrexed**. The table uses a color-coded scheme to indicate the severity and frequency of each ADR, providing a clear visual representation of how common and serious these reactions are. This helps in quickly identifying high-risk side effects and their prevalence among patients.

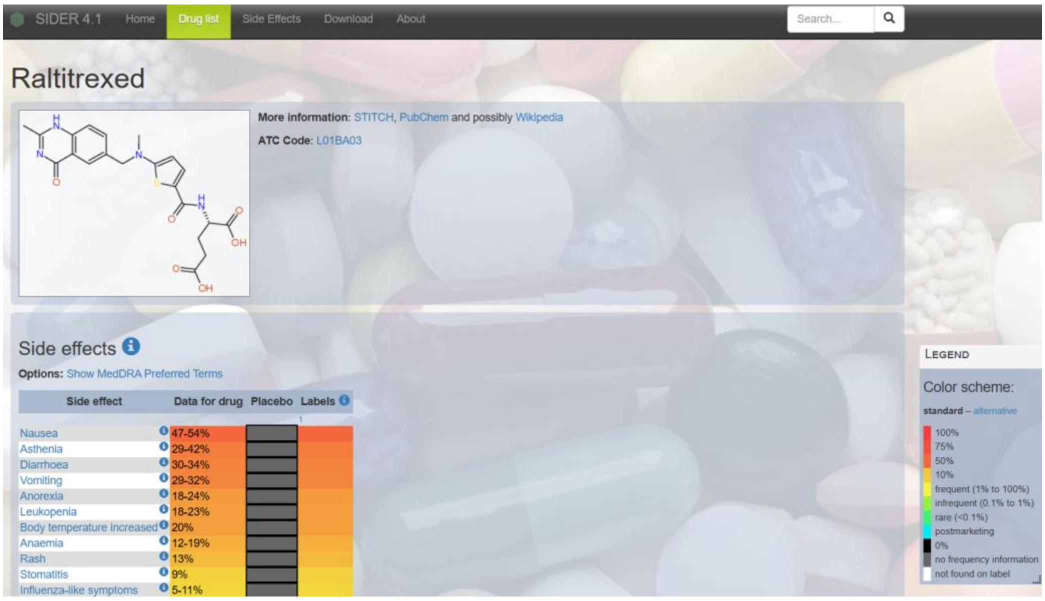

3. **Use PharmGKB to retrieve variant information related to these drugs.**

Navigating PharmGKB: The ATC classifications can be accessed via the search bar on PharmGKB's main page.

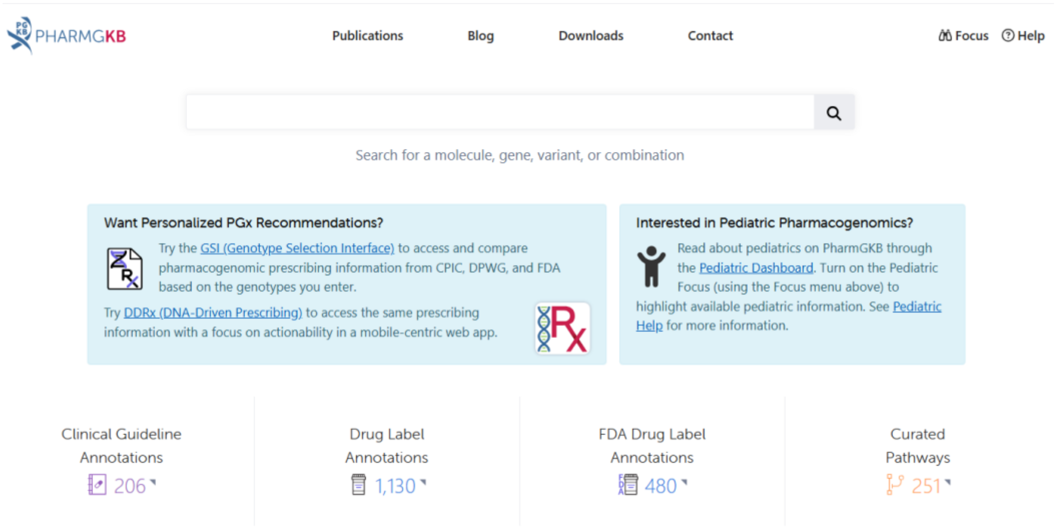

Searching for ATC Codes: After entering the L01BA code, “Folic Acid Analogues (L01BA)” is selected from the dropdown list as the first example.

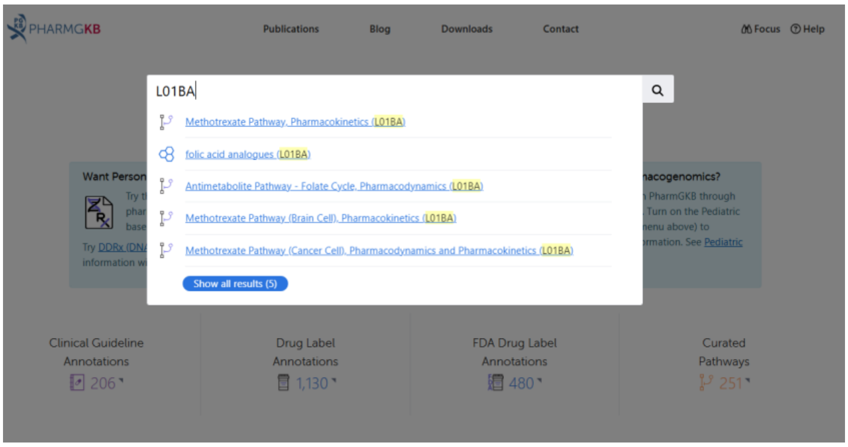

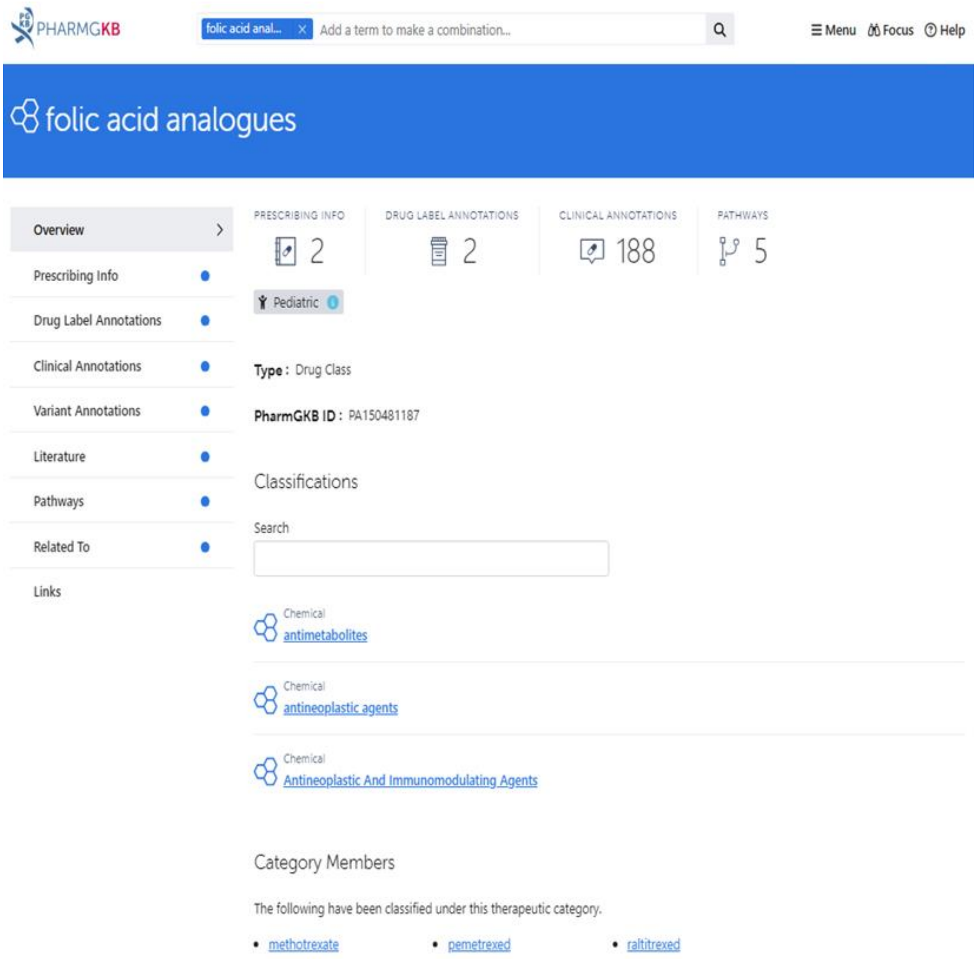

Selecting individual drugs: PharmGKB displayed only drugs from the **L01BA** category. The following snapshot provides details on the drug **Raltitrexed.**

Finding Variant Information: On this new page, variant information for 'Raltitrexed' can be found in a section located in the left menu bar.

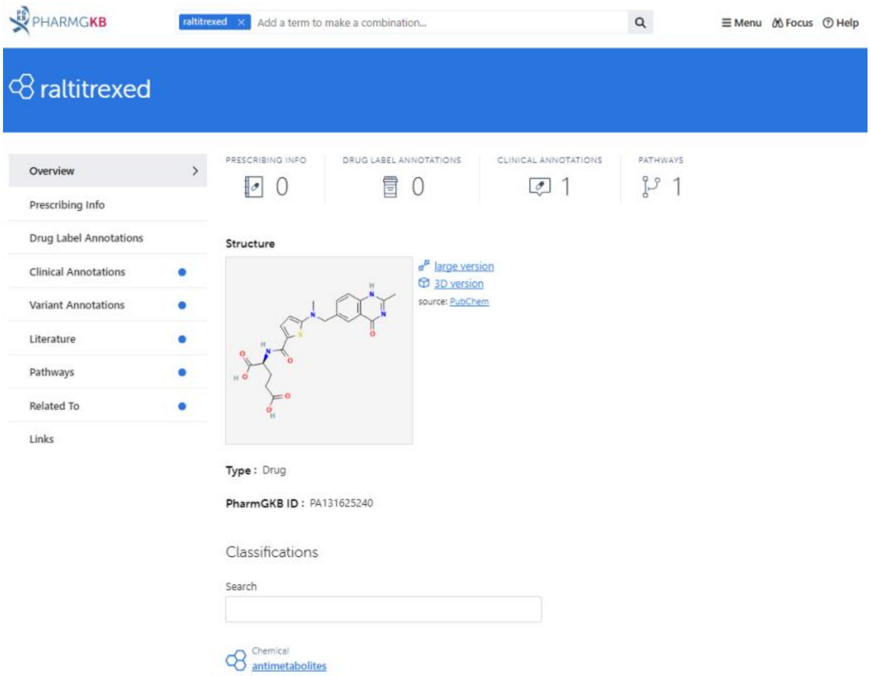

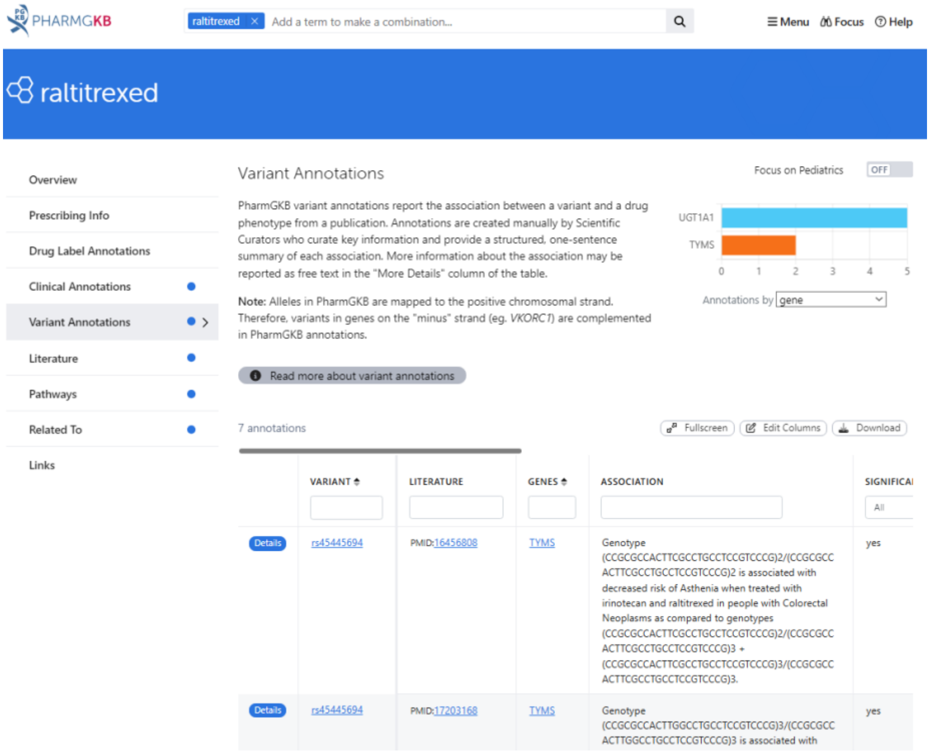

We repeated the same process for other drugs too and obtained their snapshots and also noted the time taken by this process.

4. Using RepurposeDrugs to analysis drug indications for drugs associated with L01BA

Next we copied each of seven drugs belonging to **L01BA** to get indications information from RepurposeDrugs. We copied each drug and pasted in RepurposeDrugs 'select drugs' drop down (shown in below snapshot) as RepurposeDrugs does not allow search by ATC codes. RepurposeDrugs also preselected other drugs along with these seven drugs however we manually marked at left to show the seven drugs.

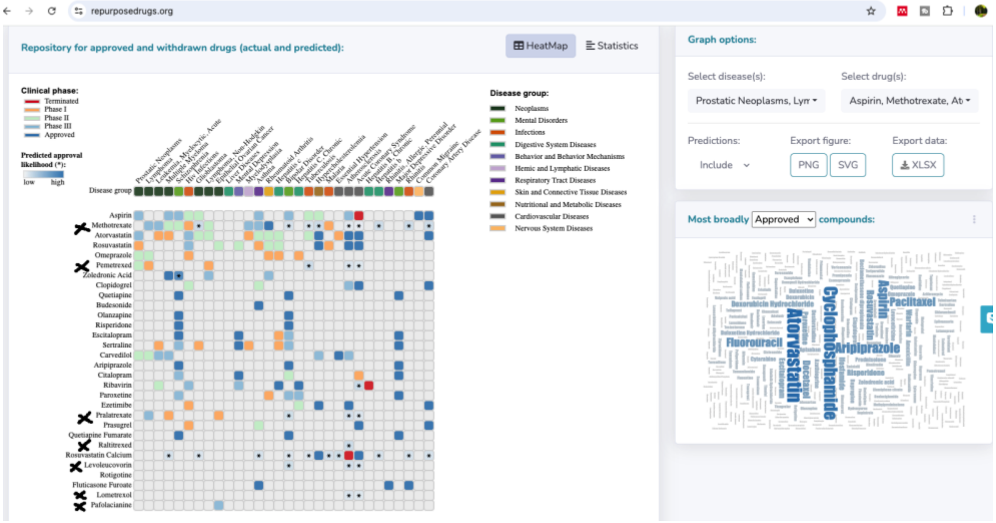
repurposedrugs.org
Repository for approved and withdrawn drugs (actual and predicted):
HeatMap
Statistics
Clinical phase:
Terminated
Phase I
Phase II
Phase III
Approved
Predicted approval likelihood (*):
low
high
Disease group
Aspirin
Methotrexate
Atorvastatin
Rosuvastatin
Omeprazole
Pemetrexed
Zoledronic Acid
Clopidogrel
Quetiapine
Budesonide
Olanzapine
Risperidone
Escitalopram
Sertraline
Carvedilol
Aripiprazole
Citalopram
Ribavirin
Paroxetine
Ezetimibe
Pralatrexate
Prasugrel
Quetiapine Fumarate
Raltitrexed
Rosuvastatin Calcium
Levoleucovorin
Rotigotine
Fluticasone Furoate
Lometrexol
Pafolacianine
Disease group:
Neoplasms
Mental Disorders
Infections
Digestive System Diseases
Behavior and Behavior Mechanisms
Hemic and Lymphatic Diseases
Respiratory Tract Diseases
Skin and Connective Tissue Diseases
Nutritional and Metabolic Diseases
Cardiovascular Diseases
Nervous System Diseases
Graph options:
Select disease(s):
Prostatic Neoplasms, Lym
Select drug(s):
Aspirin, Methotrexate, At
Predictions:
Include
Export figure:
PNG
SVG
Export data:
XLSX
Most broadly
Approved
compounds:
Cyclophosphamide
Atorvastatin
Aripiprazole
Paclitaxel
Fluorouracil
Aspirin
Doxorubicin Hydrochloride
Risperidone

Finally we Used PGxDB to retrieve variant information related to these drugs.
Accessing PGxDB: In PGxDB, users can find the ATC search bar in the "ATC Classification Hierarchy Browser" section located under "ATC Codes" in the top menu bar.

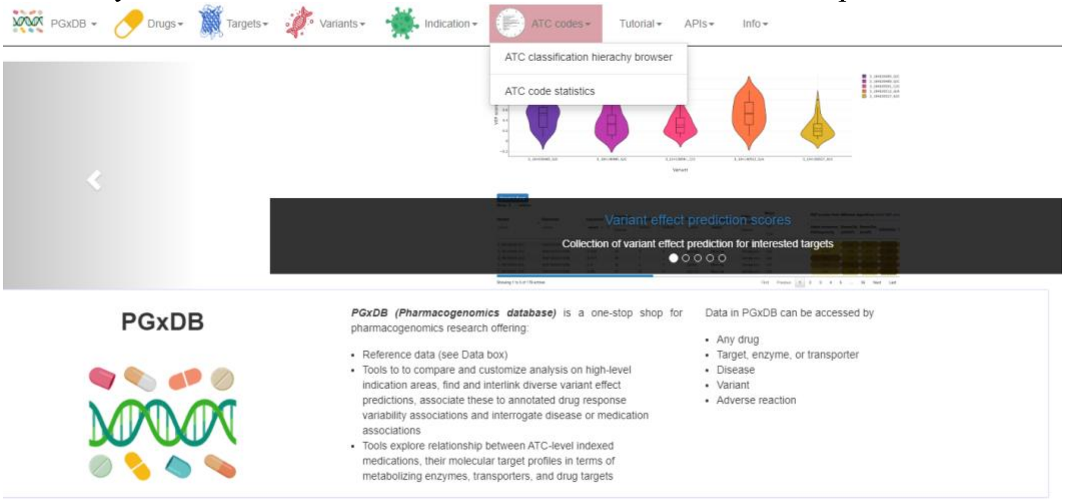

Searching for ATC codes: The L01BA code is searched, returning related entries. "L01BA - Folic acid analogues" is selected as a sample for the first example.

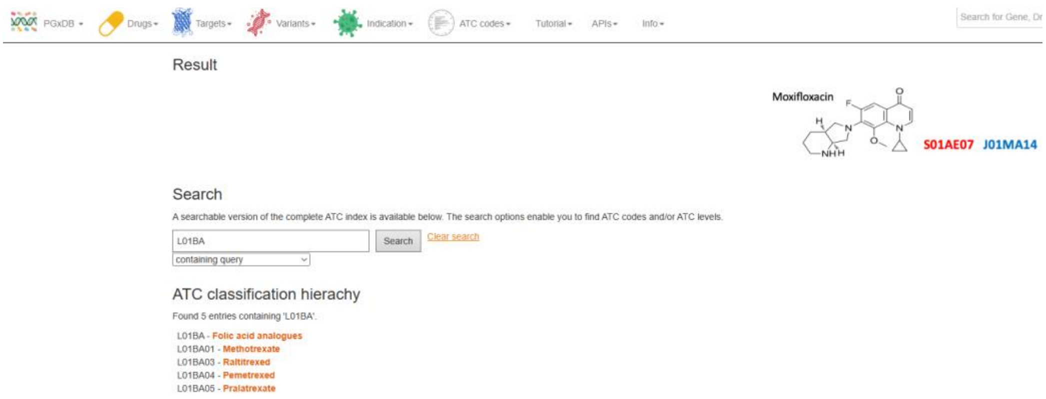

Upon clicking at 'L01BA -Folic acid analogues', PGxDB opened listed drugs, targets and indicators both in tabular and tripartite network visualization.

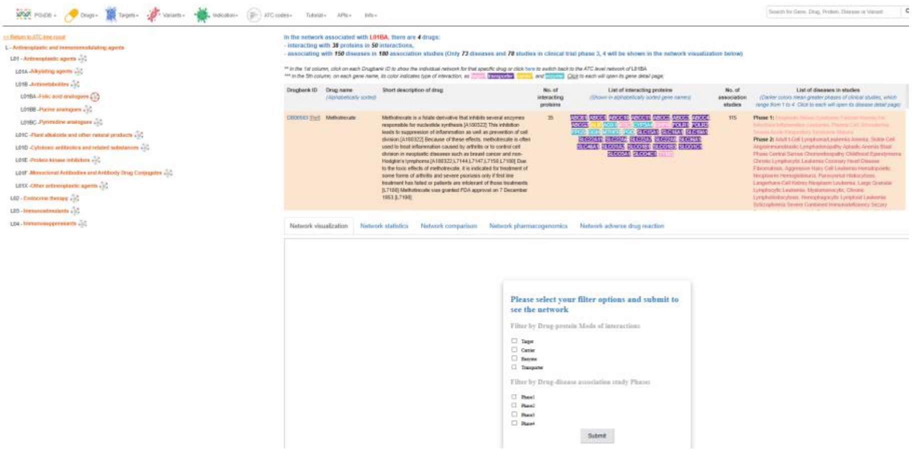

In the **tripartite network visualization** shown below, three key entities are represented: **drugs** (pill-shaped), **targets** (circular-shaped), and **disease indications** (triangular-shaped). Each entity type is color-coded based on its classification, enhancing clarity. The links between these entities are also color-coded, reflecting details such as clinical phase and target interaction types.

Users can interact with the visualization through **mouse clicks**, applying various filters and exploring the data in an intuitive manner. This network visualization not only helps users understand the **polypharmacological effects** between different drugs but also highlights **shared disease indications** among drugs, promoting opportunities for **drug repurposing**.

When users click on a **drug (pill)**, a popup opens displaying its **toxicities and physiochemical properties**. Similarly, clicking on **target nodes** provides detailed information about their interactions and associated data.

Integrated Information Access:

PGx allows users to access **targets, toxicities, adverse drug reactions (ADRs),** and **variant information** for queried ATC codes and their compounds, all on different sections of a single page. This integration showcases PGx's efficiency in **consolidating pharmacogenomics data**, streamlining the user experience.

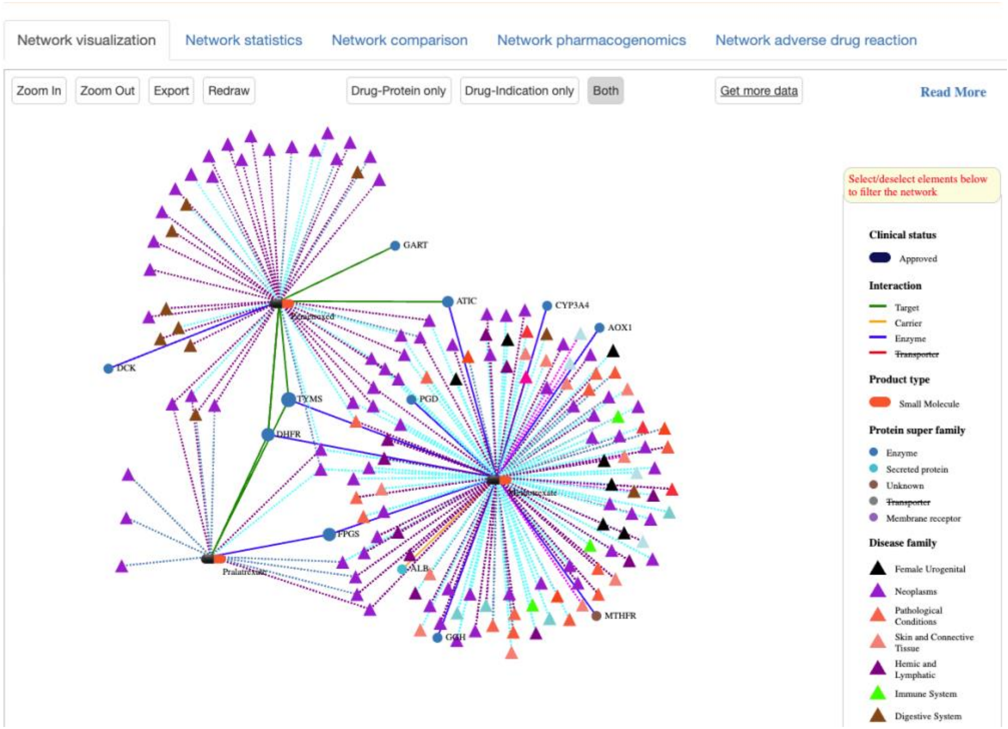

ADRs available at PGxDB for L01BA code

Network visualization | Network statistics | Network comparison | Network pharmacogenomics | Network adverse drug reaction

Drug adverse reaction of drugs in the network of L01BA
(Drug names are listed in alphabetical order; Frequencies are listed in descending order)

Read More

Export to Excel

Drugbank ID	Drug name	Side effect (SE)	SE Definition	Frequency (in percentage)
DB00293	Raltitrexed	Mouth ulceration	An inflammatory lesion on the mucosal surface of the oral cavity.	2

Drugbank ID	Drug name	Side effect (SE)	SE Definition	Frequency (in percentage)
DB00642	Pemetrexed	Agranulocytosis	NA	38
		Skin exfoliation	Shedding of the outer layer of skin or mucosal tissue.	22
		Creatinine renal clearance decreased	NA	18
		Neuropathy	A disorder affecting the cranial nerves or the peripheral nervous system. It manifests with pain, tingling, numbness, and muscle weakness. It may be the result of physical injury, toxic substances, viral diseases, diabetes, renal failure, cancer, and drugs.	17
		Mood alteration NOS	A change in disposition or state of mind.	14
		Mood swings	A condition of frequent mood changes associated with excessive emotional reactions.	14
		Alanine aminotransferase increased	An increase in the level of alanine aminotransferase in the blood.	10
		Febrile neutropenia	Neutropenia associated with fever, the latter indicating the presence of an infection.	9
		Aspartate aminotransferase increased	An increase in the level of aspartate aminotransferase in the blood.	8
		Mucosal inflammation	Inflammation of the mucous membranes.	7
		Odynophagia	Pain upon swallowing.	7
		Oesophagitis	An acute or chronic inflammatory process affecting the esophageal wall.	7
		Thrombosis	The formation of a blood clot in the lumen of a vessel or heart chamber; causes include coagulation disorders and vascular endothelial injury.	7
		Blood creatinine decreased	NA	5
		Creatinine low	NA	5
		Myocardial ischaemia	A disorder of cardiac function caused by insufficient blood flow to the muscle tissue of the heart. The decreased blood flow may be due to narrowing of the coronary arteries, to obstruction by a thrombus, or less commonly, to diffuse narrowing of arterioles and other small vessels within the heart. Severe interruption of the blood supply to the myocardial tissue may result in necrosis of cardiac muscle (myocardial infarction).	3
DB00293	Raltitrexed	Mouth ulceration	An inflammatory lesion on the mucosal surface of the oral cavity.	2

Variant information in the network of L01BA code

Network visualization | Network statistics | Network comparison | Network pharmacogenomics | Network adverse drug reaction

Read More

Export to Excel

Clinical PGx data (Variant drug response)
◉ Variant-based (only)

Burden data
○ Gene-based ○ Variant-based

Show 2 entries

Search:

Drug name (select)	Gene name (select)	MOA (select)	Variant/Haplotypes	PMID	Phenotype category (select)	Significance (select)	Alleles	P_Value (select)
Methotrexate	DHFR	Target	rs1650697	PMID:33501733	Metabolism/PK	no	G	= 0.337
Methotrexate	DHFR	Target	rs1643650	PMID:25084201	Efficacy	no	CC + CT	= 0.026

Showing 1 to 2 of 384 entries

First Previous 1 2 3 4 5 ... 192 Next Last

Conclusions on comparisons:

Following is the time taken to analyze data for L01BA code.

1. **DrugBank:**
   - Time taken: 189 seconds (27 seconds per drug x 7 drugs)
   - Effort: Users needed to search for each drug individually, gather information on drug-targets and toxicities, and manually track the data for each drug.
2. **SIDER:**
   - Time taken: 120 seconds
   - Effort: Users had to search for each drug, browse through the correct ATC code, and manually select and analyze adverse drug reactions for each drug.
3. **PharmGKB:**
   - Time taken: 23 seconds
   - Effort: Users only needed to select relevant drugs from the L01BA code and extract pharmacogenomic data.

PGx:

- Time taken: 30 seconds
- Effort: PGxBD provided all relevant information about the selected ATC code in one streamlined interface. It saved significant time by integrating multiple data points (drug-targets, ADRs, pharmacogenomics) into a single platform.

This time reduction highlights PGxBD's efficiency, offering a centralized platform where users don't need to navigate multiple databases or have specialized knowledge of each. Furthermore, PGxDB displays much of the information through **easy-to-understand visualizations**, enhancing user experience and reducing data interpretation time.
